# Pharmacological activation of SIRT6 suppresses progression of head and neck and esophageal squamous cell carcinoma by modulation of cellular metabolism and protein translation

**DOI:** 10.1038/s41419-025-07959-5

**Published:** 2025-10-16

**Authors:** Talal Ben Lulu, Dinesh Babu Manikandan, Yaniv Pevzner, Menachem Sklarz, Sarah Adi Eisdorfer, Samaa Awad, Sooraj Mathukkada, Divyasree Marripati, Monica Wainer, Liana Shimshilashvili-Kleiner, Ehud Ohana, Idan Cohen, Jian Zhang, Shatha S. Alassam, Barak Rotblat, Hai Wang, Dexin Kong, Ofir Cohen, Debra Toiber, Moshe Elkabets

**Affiliations:** 1 The Shraga Segal Department of Microbiology, Immunology, and Genetics, Ben-Gurion University of the Negev, Beer-Sheva, Israel; 2 Department of Clinical Biochemistry and Pharmacology, Faculty of Health Sciences, Ben-Gurion University of the Negev, Beer-Sheva, Israel; 3 The School of Brain Sciences and Cognition, Ben Gurion University of the Negev, Beer-Sheva, Israel; 4 State Key Laboratory of Medical Genomics, Ruijin Hospital, Shanghai Jiao Tong University School of Medicine, Shanghai, China; 5 Department of Pharmaceutical and Artificial-Intelligence Sciences, Institute of Medical Artificial Intelligence, Shanghai Jiao Tong University School of Medicine, Shanghai, China; 6 Department of Life Sciences, Ben-Gurion University of the Negev, Beer-Sheva, Israel; 7 CAS Key Laboratory for Biomedical Effects of Nanomaterials & Nanosafety, CAS Center for Excellence in Nanoscience, National Center for Nanoscience and Technology, University of Chinese Academy of Sciences, Beijing, China; 8 Tianjin Key Laboratory of Technologies Enabling Development of Clinical Therapeutics and Diagnostics, School of Pharmacy, Tianjin Medical University, Tianjin, China

**Keywords:** Head and neck cancer, Cancer metabolism, Cell signalling

## Abstract

Sirtuin 6 (SIRT6), a NAD+-dependent histone deacetylase, has been shown to function as a tumor suppressor in several cancer types, including squamous cell carcinoma of the head and neck and the esophagus (HNSCC and ESCC). However, the potential of therapies involving the activation of SIRT6 in HNSCC and ESCC remains unexplored. In this work, we investigated the therapeutic potential and mechanisms of action of the allosteric SIRT6 activator MDL-800 in HNSCC and ESCC models. MDL-800 treatment inhibited the proliferation and migration of HNSCC and ESCC cell lines in vitro, and delayed tumor growth in cell-derived xenograft models, in vivo. Mechanistically, global H3K9ac acetylation profiling and protein arrays demonstrated that MDL-800 treatment potently inhibits glucose metabolism and protein translation by inhibiting mTOR signaling, E2F-related G1/S transcription, ribosomal protein S6 (S6) and 4E-BP1 activity. This inhibition of mTOR induces a compensatory feedback loop activating IGF-1R/INSR signaling, limiting the anti-tumor activity of MDL-800. Combination of MDL-800 with the alpha-specific PI3K inhibitor (BYL719/Alpelisib) disrupted this feedback loop activation and resulted in a synergistic anti-proliferative effect. In vivo, the combined treatment of MDL-800 and BYL719 resulted in a prolonged anti-tumor response. Overall, our study identifies the molecular mechanism underlying SIRT6 activation in HNSCC and ESCC and highlights the therapeutic potential of SIRT6 activators, alone or in combination with PI3K inhibitors in cancers where SIRT6 is downregulated or serves as a tumor suppressor.

## Introduction

Squamous cell carcinoma (SCC) arises from squamous epithelial cells in mucosal tissues and can affect the head and neck area (e.g., oral cavity, pharynx, larynx, etc.), giving rise to head and neck squamous cell carcinoma (HNSCC), and the esophagus, leading to esophageal squamous cell carcinoma (ESCC) [1, 2]. Transformation from normal epithelium to SCC, the predominant subtype of both head and neck and esophageal cancer, begins with epithelial cell hyperplasia, followed by dysplasia, carcinoma in situ, which is ultimately followed by invasive carcinoma [1, 3]. In line with their histopathological similarity, HNSCC and ESCC share several key characteristics, such as their primary risk factors – alcohol consumption and tobacco usage, nutritional factors, and ethnicity [1, 4]. In addition, HNSCC and ESCC share similar molecular characteristics, such as mutations in the genes TP53, PIK3CA, CCND1, EGFR, and others [2, 5]. Since HNSCC and ESCC share several molecular targets, similar therapies are being investigated for both diseases [6].

HNSCC is the sixth most common type of cancer, with a 5-year survival rate of 66%, while ESCC is the eighth most common, with a 5-year survival rate that ranges from 15% to 25% [1, 4, 7]. Collectively, these cancer types contribute to nearly 1.5 million new cases and 900,000 deaths worldwide annually, and the incidence is expected to continue and rise [1, 4]. Current treatment for HNSCC and ESCC includes surgery followed by radiotherapy, chemotherapy, immunotherapy, and targeted therapy [8, 9]. However, the emergence of resistance to chemotherapy, radiotherapy, and some targeted therapies often occurs, resulting in malignancy progression and mortality [10, 11]. The increasing incidence, together with the inadequate clinical outcomes of current therapies, stresses the importance of identifying novel therapeutic targets for HNSCC and ESCC.

Sirtuin 6 (SIRT6) is a member of the Sirtuins, a family of nicotinamide adenine dinucleotide (NAD + )-dependent class III histone deacetylases (HDACs) [12]. SIRT6 possesses several enzymatic activities, including fatty acid deacylation, mono-ADP-ribosylation, and deacetylation of non-histone and histone proteins [13], with the latter being the most extensively studied. These post-translational modifications make the enzymatic activity of SIRT6 valuable to the integrity of multiple cellular processes and enable SIRT6 to regulate various physiological and pathological processes [12]. Notably, not all SIRT6 functions are dependent on its enzymatic activity [14]. Both through its enzymatic activity and independently, SIRT6 was shown to regulate various cellular processes, including metabolism, glucose homeostasis, gene expression, inflammation, genomic stability, DNA damage repair, and aging [15]. As a result of its involvement as a regulator of multiple cellular processes, SIRT6 was shown to play a major role in the development and progression of multiple cancer types [12, 16]. Interestingly, the role of SIRT6 in cancer was shown to be tissue-specific, acting as a tumor promoter in some types of cancer while acting as a tumor suppressor in others [17]. In HNSCC, SIRT6 was shown to act as a tumor suppressor, as its downregulation is associated with shorter overall patient survival [18–20]. Moreover, Choi and colleagues recently demonstrated that SIRT6 knock-out (KO) enhanced tumor development in mice that were exposed to a carcinogen compared to their wild-type (WT) counterparts and showed that this tumor-suppressive role is related to SIRT6 negative regulation of glycolysis [18].

Given the fundamental role of SIRT6 in maintaining cellular homeostasis and its involvement in several diseases, including cancer, it has emerged as an appealing target for the development of specific modulators. In the effort to identify small-molecule activators of SIRT6, Huang and colleagues reported the identification of the cellularly active allosteric activator MDL-800 [21]. MDL-800 allosterically binds to SIRT6, and by increasing the binding affinities of its cofactor and substrates, it activates its deacetylate activity and catalytic efficiency. In hepatocellular carcinoma (HCC) cells, MDL-800 treatment was shown to decrease the acetylation levels of two well-known SIRT6 substrates, H3K9ac and H3K56ac, and to consequently inhibit their proliferation by inducing cell-cycle arrest in hepatocellular carcinoma and in non-small cell lung cancer (NSCLC) [21, 22]. Here, we hypothesized that increased activation of SIRT6 in HNSCC and ESCC would induce tumor growth delay, and thus, we sought to investigate MDL-800 as a potential therapeutic target for these malignancies.

## Materials and methods

### Cell lines and chemical compounds

The human HNSCC cell lines SNU-1076 (Korean Cell Line Bank), CAL33 (German Collection of Microorganisms and Cell Cultures (DSMZ)), HSC-2 (Health Sciences Research Resources Bank), CAL27, Detroit562, and FaDu (American Type Culture Collection) and ESCC cell line KYSE-180 (DSMZ), were purchased from the mentioned commercial vendors. All cell lines were maintained at 37° in a humidified atmosphere at 5% CO_2_ in Dulbecco’s Modified Eagle’s medium (DMEM) or RPMI-1640 medium supplemented with 1% L-glutamine 200 mM, 100 units of penicillin and streptomycin 10% fetal bovine serum (FBS).

### In vivo experiments

NOD.Cg-Prkdc^scid^ Il2rg^tm1Wjl^/SzJ (NOD SCID gamma; NSG) mice were purchased from The Jackson Laboratory. Mice were housed in air-filtered laminar flow cabinets with a 12/12-hour light/dark cycle and were fed food and water ad libitum. Mice were maintained and treated according to the institutional guidelines of Ben-Gurion University of the Negev, and the experiments were approved by the Institutional Animal Care and Use Committee [IL-57-12-2022E]. In accordance with the approved protocol, the maximum tumor size permitted by the ethics committee was 1000 mm³. This limit was not exceeded at any point during the study. To generate cell-derived xenografts, KYSE-180, HSC-2, FaDu and CAL-27 cells (10 × 10^6^, 5 × 10^6^, 4 × 10^6^ and 5 × 10^6^ cells, respectively) were suspended in 60 μl of Phosphate Buffered Saline (PBS) and injected subcutaneously into the backs of 6–8 weeks old female NSG mice. When tumors reached the volume of 100 mm3, mice were randomized into 2 or 4 groups of 4-6 mice per group, depending on the experiment. In all In vivo experiments, MDL-800 (80 mg/kg) was dissolved in 5% Dimethyl Sulfoxide (DMSO), 30% Polyethylene glycol 300 (PEG300), and 65% PBS and administered every other day by intraperitoneal injection (IP), and BYL719 (25 mg/kg) was dissolved in 5% carboxymethyl cellulose (CMC) and administered daily by oral gavage. Drug-treated mice received MDL-800, BYL719, or both, depending on the experiment. Vehicle-treated mice received 5% DMSO, 30% PEG300, 65% PBS, 5% CMC, or both, depending on the experiment. Tumors were measured with a digital caliber twice a week, and tumor volumes were calculated according to the formula: Tumor volume = (L × W × W), where W stands for tumor width and L for tumor length. At the end of the experiments, animals were euthanized with CO2, and the tumors were harvested, fixed in 4% paraformaldehyde (PFA) overnight, and stored in 70% ethanol for investigation. Measurements of tumor volumes are plotted as means ± SEM.

### Statistical analysis

Statistical analysis was performed using GraphPad Prism software version 9, and results are presented as means ± SEM. For comparisons between two groups, P values were calculated using unpaired t-test. For comparisons between three or more groups, P values were calculated using one-way ANOVA with Tukey’s multiple comparison test (*p < 0.05, **p < 0.01, ***p < 0.001). Statistical analysis for protein and RTK arrays was performed using unpaired multiple t-tests with false discovery rate (FDR) control, applying the two-stage step-up method by Benjamini, Krieger, and Yekutieli. Statistical analysis for comparing growth curves from in vivo experiments was performed using CGGC permutation tests developed by Walter& Elisa Hall bioinformatics - Institute of Medical Research - http://bioinf.wehi.edu.au/software/compareCurves/index.html [23].

## Results

### MDL-800 treatment inhibits the progression of HNSCC and ESCC in vitro and in vivo

**Fig. 1:**
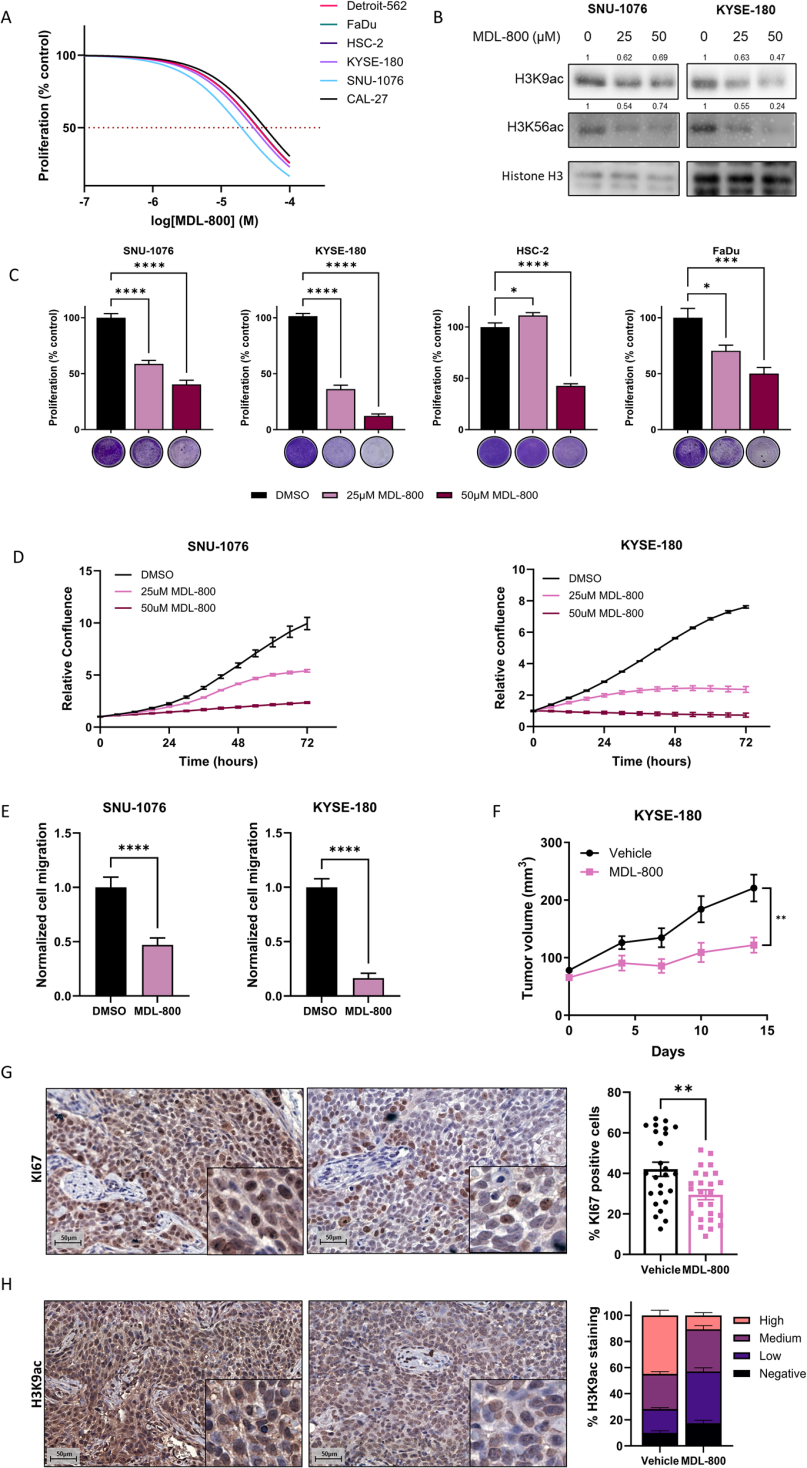
Characterization of the anti-tumor effect of MDL-800 in HNSCC and ESCC. **A** Dose-response curve of the indicated cell lines following 72 hours of treatment with increasing concentrations of MDL-800 (0–100 μM), *N* = 3. **B** Western blot analysis for H3K9ac and H3K56ac in SNU-1076 and KYSE-180 cells treated with DMSO, 25 µM MDL-800, or 50 µM MDL-800 for 48 hours. Histone H3 served as a loading control. Relative protein levels were calculated relative to Histone H3 loading control and presented as fold change from the control sample. **C** SNU-1076, KYSE-180, HSC-2, and FaDu cells were treated with DMSO, 25 μM or 50 μM of MDL-800 for 72 hours. Cell viability was determined using crystal violet staining. Error bars indicate SE, *N* = 3. **D** Growth rate of SNU-1076 and KYSE-180 cells treated with DMSO, 25 μM or 50 μM of MDL-800 for 72 hours. Cell confluence was monitored by a live cell imager every 6 hours. Error bars indicate SE, N = 3. Data represents a representative experiment from two independent experiments. **E** Following 24-hour treatment of DMSO or 25uM, SNU-106 and KYSE-180 cells were seeded in a trans-well and allowed to migrate for 24 hours. The number of migrated cells was quantified after fixation and staining. Error bars indicate SE, *N* = 3. **F** Tumor growth kinetics of KYSE-180 derived tumors in NSG mice (*n* = 4) treated with vehicle or MDL-800 (80 mg/kg). Statistical significance was calculated by CGGC permutation test (*p < 0.05, **p < 0.01, ***p < 0.001, ****p < 0.0001). **G** KI67 IHC staining of tumors from vehicle and MDL-800-treated mice. The percentage of positive cells was calculated and presented in a bar graph. Error bars indicate SE. Statistical significance was calculated using an unpaired t-test (*p < 0.05, **p < 0.01, ***p < 0.001, ****p < 0.0001). **H** H3K9ac IHC staining of tumors from vehicle and MDL-800-treated mice. The percentage of relative staining levels was calculated and presented in a bar graph. Error bars indicate SE.

To evaluate the sensitivity of ESCC and HNSCC cell lines to MDL-800 in vitro, we started by measuring the half-maximal inhibitory concentration (IC_50_) of MDL-800 in human HNSCC (HSC-2, CAL33, CAL27, FaDu, Detroit-562, SNU-1076) and ESCC cell line (KYSE-180). A variable response to MDL-800 was detected, with IC_50_ values ranging between 19.71 µM and 58.6 µM (Fig. 1A, Supplementary Fig. 1A). We noted that the tumor cell lines responded to MDL-800 in a dose-dependent manner, and the highest sensitivity to MDL-800 was observed in HNSCC cell line SNU-1076 and the ESCC cell line KYSE-180, with IC_50_ values of 19.71 µM and 29.59 µM, respectively. Based on the IC_50_ values and the proliferation assay, we decided to focus mostly on SNU-1076 and KYSE-180 cell lines for further studies. To ensure the on-target effect of MDL-800, we measured the acetylation levels of SIRT6 substrates H3K9ac and H3K56ac in two cell lines treated with 25 µM and 50 µM [22], and observed that MDL-800 treatment decreased both H3K9ac and H3K56ac acetylation levels (Fig. 1B). We next confirmed the anti-proliferative activity in multiple cells lines by treating them with 25 µM and 50 µM of MDL-800 and performing a proliferation assay, as these concentrations were previously reported to effectively inhibit cell viability [22]. Notably, 25 µM MDL-800 treatment significantly inhibited the proliferation of 3 out of 4 cell lines, while 50 µM significantly inhibited the proliferation of all tested cell lines (Fig. 1C). Moreover, live imaging proliferation assay showed that SNU-1076 and KYSE-180 cell lines displayed substantial growth inhibition when treated with 25 µM and 50 µM MDL-800 (Fig. 1D). Further analysis showed that MDL-800 induced a cytostatic effect with G1-phase cell cycle arrest in the KYSE-180 cell line without inducing apoptosis (Supplementary Fig. 1 B, C). We next evaluated the efficacy of MDL-800 in inhibiting cell migration in vitro by performing a transwell migration assay and found that treatment of SNU-1076 and KYSE-180 tumor cells with 25 µM of MDL-800 resulted in a significant decrease in cell migration compared to treatment with DMSO (Fig. 1E).

To evaluate the efficacy of MDL-800 in vivo, we created a cell line-derived xenograft (CDX) model by implanting the highly MDL-800-sensitive KYSE-180 tumor cells and modestly sensitive HSC-2 and FaDu tumor cells into NSG mice and treated them with vehicle or MDL-800 (80 mg/kg) every other day by intraperitoneal (i.p) injection. MDL-800 treatment induced a significant growth delay of KYSE-180 tumors when compared to vehicle treatment (Fig. 1F). Immunohistochemistry (IHC) staining of KYSE-180 tumor tissue showed that treatment of MDL-800 reduced tumor cell proliferation, as measured by staining of the proliferation marker Ki67 (Fig. 1G) and decreased the staining of the SIRT6 substrate H3K9ac (Fig. 1H). Similar results, but less robust, were observed in HSC-2 and FaDu tumor-bearing mice. Although MDL-800 inhibited the growth of HSC-2 and FaDu tumors in mice, tumors quickly started to progress at a similar rate to the vehicle-treated group (Supplementary Fig. 1D, E). Overall, these results indicate that the activation of SIRT6 with MDL-800 has anti-proliferative activity in HNSCC and ESCC cell lines in vitro and in vivo.

### SIRT6 activation results in broad cellular dysregulation in HNSCC and ESCC

**Fig. 2:**
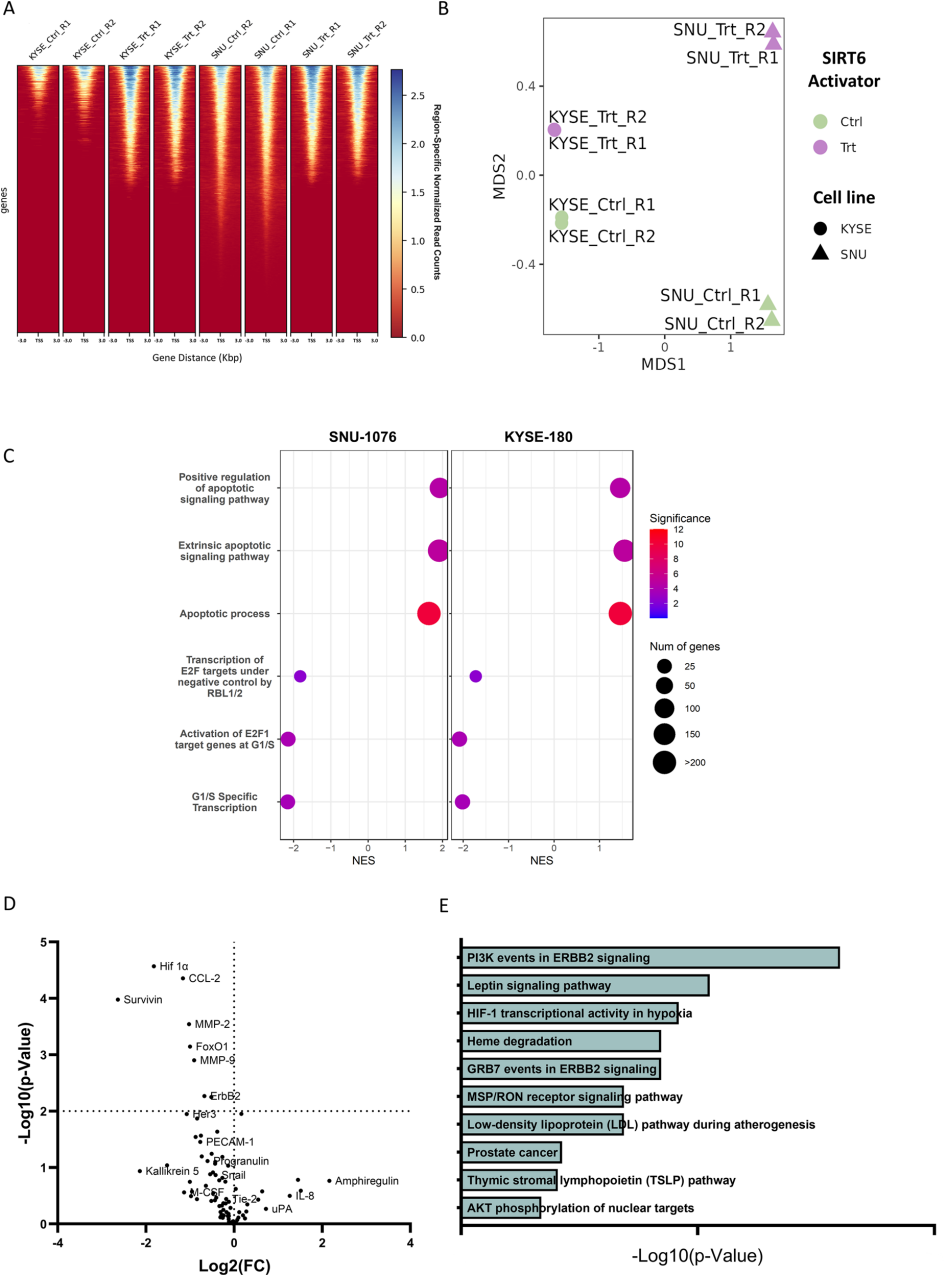
The SIRT6 activation cell state changes reflected in altered chromatin and protein expression suggesting induction of apoptosis, inhibition of cell-cycle transcription, and broad signaling pathways dysregulation in HNSCC and ESCC. **A** The tornado plot visualizing binding intensity near the transcription start sites (TSS) across the global profiles of H3K9ac acetylation with (CUT&RUN) sequencing with for SNU-1076 (SNU) and KYSE-180 (KYSE) cells treated with MDL-800 (Trt) or DMSO control (Ctrl) in two replicates. **B** Multidimensional scaling (MDS) of the genome-wide CUT&RUN patterns, reveal high reproducibility among replicates and the two main sources of variability - with MDS1 reflecting the differential representation of H3K9ac peaks between the two cell-lines and MDS2 reflecting differential peaks between MDL-800 treatment and control. **C** Gene Set Enrichment Analysis (GSEA) of the most pronounces pathways that are significantly dysregulated in both cell lines under SIRT6 treatment include induction of apoptotic signaling, as reflected with positive Normalized Enrichment Score (NES) across three apoptosis-related Gene Ontology Biological Process gene sets and inhibition of E2F and G1/S cell-cycle transcription and reflected with negative NES across three related gene sets. The GSEA significance is color-coded as -log10 (*p*-value) and the size of each GSEA dot represent the number of genes in the set. **D** Volcano plot showing the altered expression of oncogenic proteins for SNU-1076 and KYSE-180 cells treated for 48 hours with 25 µM MDL-800. Statistical significance calculated using unpaired multiple t-tests with FDR control. **E** Bar graph showing the 10 most enriched downregulated pathways sorted by p-value ranking in SNU-1076 and KYSE-180 cells following 48 hours MDL-800 treatment.

To explore the mechanism underlying the anti- proliferative effect of MDL-800 in KYSE-180 and SNU-1076 cell lines, we profiled the global acetylation of H3K9 in cells treated with either DMSO or MDL-800. Tumor cells were treated for 48 hours, after which their nuclei were extracted, incubated with an H3K9ac-specific antibody or an IgG control and subjected to Cleavage under targets & release using nuclease (CUT&RUN), followed by DNA sequencing to identify genome-wide changes in H3K9 acetylation patterns (Fig. 2A). Global profiles of H3K9ac peaks reveal broad changes between SIRT6 activation (MDL-800) and DMSO control reflected in both KYSE-180 and SNU-1076 cell lines, as visualized by Multidimensional scaling (MDS), with MDS1 broadly reflecting the differential representation of H3K9ac peaks between the cell-lines and MDS2 reflecting differential peaks between treatment and control (Fig. 2B).

To assess the epigenetic impact of MDL-800 and its potential role in modulating gene expression, we performed a global analysis of differential peak representation between MDL-800-treated cells and control while mapping peaks to genes in their immediate proximity (See Methods, Supplementary Table S1 with listed differentially affected genes). Next, to explore the biological processes and molecular mechanisms underlying the effects of MDL-800, we performed Gene Set Enrichment Analysis (GSEA) based genome-wide on the Fold-Change of peak intensity under treatment (See Methods). The analysis revealed broad chromatin dysregulation across both KYSE-180 and SNU-1076 cell lines (Supplementary Table S2 with listed gene sets enriched or depleted under MDL-800 treatment). Among the most pronounced biological processes that were similarly disrupted in KYSE-180 and SNU-1076, we observed robust activation of apoptosis and inhibition of E2F and G1/S cell-cycle transcription (Fig. 2C).

To gain further insight into the molecular changes that occur in tumor cells following treatment with MDL-800, we profiled key proteins related to cancer using a targeted protein array. Specifically, we tested the abundance of key oncogenic proteins in SNU-1076 and KYSE-180 cell lines after treatment with 25 μM of MDL-800 or DMSO for 48 hours. Densitometric analysis of the two cell lines showed major changes in multiple oncogenic pathways, including ones related to cell migration, regulation of cell cycle, and glucose metabolism, as expected (Supplementary Fig. 2A). The volcano plot indicates the changes in specific proteins in two cell lines (Fig. 2D). Among the top down-regulated proteins, we observed HIF-1α, a known transcription factor regulated by SIRT6 [24], which we further validated using WB analysis (Supplementary Fig. 2C). Next, we performed pathway enrichment analysis to identify shared downregulated pathways using the BioPlanet 2019 database [25], and found that the proteins from the PI3K/AKT/mTOR pathway were the most downregulated (Fig. 2E, Supplementary Fig. 2B).

### SIRT6 activation inhibits mTOR and protein translation, disrupts glucose metabolism and induces AKT hyperactivation via stimulation of IGF1R/INSR in HNSCC and ESCC cell line

**Fig. 3:**
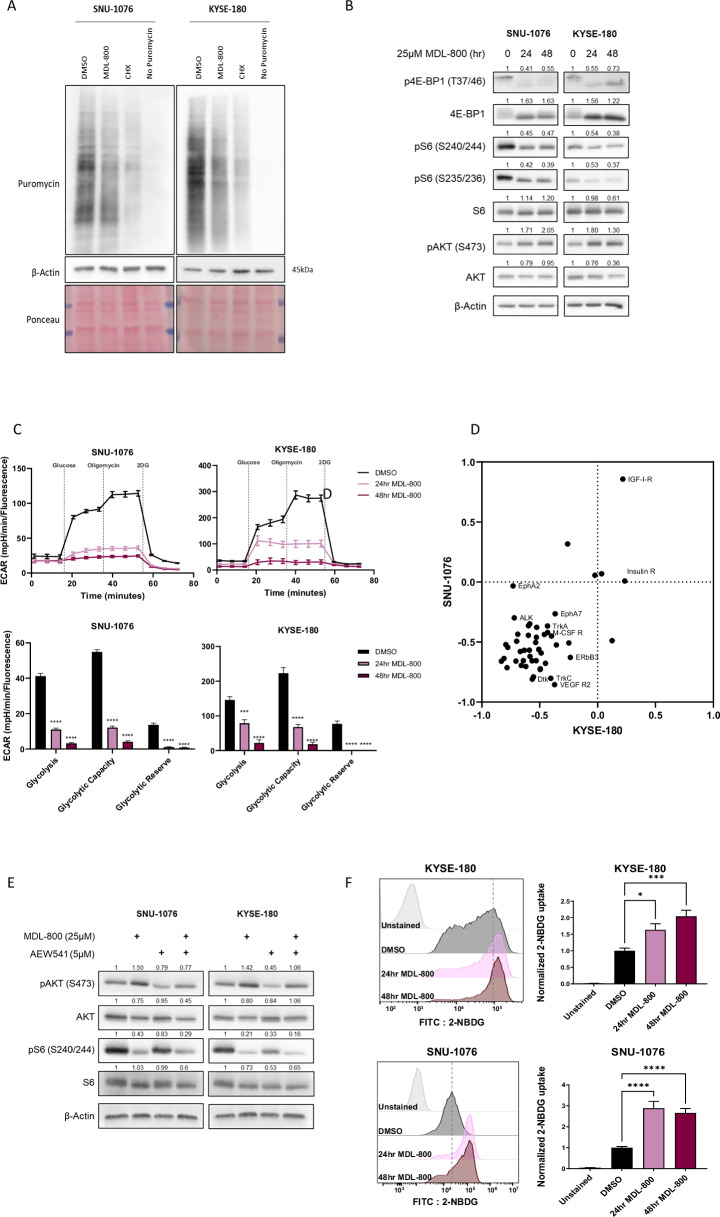
SIRT6 activation inhibits mTOR and induces AKT hyperactivation and results in disruption of translation and glucose metabolism in HNSCC and ESCC cell line. **A** Western blot analysis for SUnSET assay in SNU-1076 and KYSE-180 cells treated with puromycin for 20 minutes following 48 hours treatment of 25 µM MDL-800. Cells treated with 50 µg/ml cycloheximide (CHX) for 1 hour served as a negative control. Membranes were stained with ponceau prior to immunoblotting and β-Actin served as a loading control. Data represents a representative experiment from two independent experiments. **B** Western blot analysis for total protein and phosphorylated S6, 4E-BP1 and AKT in SNU-1076 and KYSE-180 cells treated with 25 µM MDL-800 for 48 hours. β-Actin served as a loading control. Relative protein levels were calculated relative to β-Actin loading control and presented as fold change from the control sample. Data represents a representative experiment from two independent experiments. **C** Upper panels: ECAR measurements of KYSE-180 and SNU-1076 cells treated for 24 and 48 hours with DMSO or MDL-800. Cells were treated with the following compounds: glucose for activation of glycolysis, the mitochondrial complex V inhibitor oligomycin, and the glycolysis inhibitor 2-Deoxy-D-glucose (2DG). Error bars indicate SE, *n* = 5. Data represents a representative experiment from two independent experiments. Lower panels: ECAR measurements of glycolysis, glycolytic capacity and glycolytic reserve of the experiment described in above. Error bars indicate SE. Data represents a representative experiment from two independent experiments. **D** The relative expression of each RTK was calculated for both SNU-1076 (Y-axis) and KYSE-180 (X-axis) cells treated for 48 hours with 25 µM MDL-800. RTKs that were upregulated in both cell lines are shown in the higher-right quartile; RTKs that were downregulated in both cell lines are shown in the lower-left quartile. **E** Western blot analysis for total protein and phosphorylated AKT and S6 in SNU-1076 and KYSE-180 cells treated with DMSO, 25 µM MDL-800 for 48 hours, 5 µM AEW541 for 1 hour or both. β-Actin served as a loading control. Relative protein levels were calculated relative to β-Actin loading control and presented as fold change from the control sample. Data represents a representative experiment from two independent experiments. **F** Flow cytometry analysis of 2-NBDG uptake by KYSE-180 and SNU-1076 cells treated with DMSO or 25 μM MDL-800 for 24 or 48 hours. Normalized 2-NBDG mean fluorescence intensity is shown as bar graph. Error bars indicate SE, *N* = 3. Statistical significance was calculated using one-way ANOVA (*p < 0.05, **p < 0.01, ***p < 0.001, ****p < 0.0001).

Given the broad dysregulation observed in chromatin accessibility and cellular signaling at the protein level, we next investigated the reduction in global gene expression by assessing total protein levels and measuring protein translation. Briefly, puromycin was used to label newly synthesized proteins, followed by a WB analysis to detect the total amount of translated protein. MDL-800 treatment decreased protein translation in both cell lines (Fig. 3A, Supplementary Fig. 3A). Altogether, our analysis indicates that MDL-800 has a broad effect on the tumor cells and downregulation of key elements in cancer progression.

Due to the well-established link between PI3K/AKT/mTOR pathway to translation [26] and the known roles of SIRT6 in regulating mRNA translation [27, 28], we decided to explore in detail the effect of MDL-800 on this pathway. To this end, we treated tumor cell lines with MDL-800 or DMSO for 24 and 48 hours and showed that MDL-800 substantially suppressed the phosphorylation of S6 and Eukaryotic translation initiation factor 4E-binding protein 1 (4E-BP1), both proposed to inhibit protein translation [29, 30]. To understand why mTOR is negatively regulated, we tested the levels of AMP-activated protein kinase (AMPK) and its downstream target acetyl-CoA carboxylase (ACC) by WB analysis, key negative regulators of mTOR activity [26] but no increase in AMPK activation following MDL-800 treatment was observed (Supplementary Fig. 3B). Interestingly, while a reduction of mTOR activity was detected, a rapid feedback loop activation of AKT was detected, indicated by an increase of pAKT473 (Fig. 3B). This activation of AKT following mTOR inhibition was well documented as a mechanism of resistance [31], and thus, we were interested in further understanding how MDL-800 induced the activation of AKT and if it is related to the reduction in glucose metabolism.

Next, we explored the altered cell state with respect to metabolic rewiring and glucose uptake mediate by SIRT6 [24, 32] and identified significant reductions in pathways related to the downregulation of metabolism, which suggest potential disruptions in cellular energy production and gene regulation, both of which are critical for tumor growth and survival. Based on these findings, we prioritized these pathways for further experimental validation to better understand their role in the anti-tumor effects of MDL-800. Because SIRT6 was shown to exert its anti-tumorigenic effect by attenuating glucose metabolism [18, 24], we sought to explore it in our HNSCC and ESCC cell line models. To examine the impact of MDL-800 on glycolysis, we used the Seahorse XF Analyzer to perform the Seahorse glycolysis Stress assay. Following 24 and 48 hours of treatment with MDL-800, the extracellular acidification rate (ECAR) after the addition of glucose lowered significantly, indicating cellular glycolysis was strongly inhibited, with almost complete inhibition of glycolysis after 48 hours of treatment in both KYSE-180 and SNU-1076 cell lines (Fig. 3C).

Notably, additional glycolytic parameters were inhibited following 24 and 48 hours of MDL-800 treatment. Glycolytic capacity, which is measured as the maximal ECAR measurement reached by the cells after the addition of oligomycin that inhibits oxidative phosphorylation and thus drives the cells to use glycolysis to the maximum capacity, was significantly inhibited in both cell lines. Moreover, glycolytic reserve, which measures the capability of cells to respond to energetic demands and indicates their proximity to their theoretical maximal glycolytic function, was robustly inhibited in both KYSE-180 and SNU-1076 cell lines (Fig. 3C).

To explore the mechanism by which SIRT6 activation increases AKT phosphorylation, we characterized the activation status of key receptor tyrosine kinases (RTKs) in SNU-1076 and KYSE-180 cells treated with DMSO or MDL-800 for 48 hours using phospho-protein array of 42 RTKs. The analysis revealed substantial inhibition of multiple RTKs following MDL-800 treatment, with an almost exclusive activation of the metabolic-related RTKs Insulin-like growth factor 1 receptor (IGF1-R) in both cell lines and upregulation of Insulin receptor (INSR) in KYSE-180 cells (Fig. 3D, Supplementary Fig. 3C). These results align with a previous report showing that inhibition of mTOR results in increased S473 AKT phosphorylation through activation of IGF-1R [31]. To explore if the activation of IGF-1R/IR is responsible for AKT hyperactivation, we blocked IGFR1 activity in cells using AEW541. Specifically, we co-treated SNU-176 and KYSE-180 tumor cells with MDL-800 with or without AEW541 for 48 hours and noticed that supplementation of AEW541 attenuated the MDL-800 induced-activation of AKT to its baseline levels of untreated cells (Fig. 3E). To gain further insight on glucose metabolism and activation of IGFR1, we explored if MDL-800 treatment influences glucose uptake using the fluorescent glucose analog, 2-NBDG. Flow cytometry analysis showed that MDL-800 treated cells exhibited increased glucose uptake compared to DMSO control-treated cells (Fig. 3F). These results indicate that the inhibition of glycolysis of the tumor cells following MDL-800 treatment is not induced by a reduction of glucose levels in the cells, but rather by disruption of glycolysis machinery in the cells, and the uptake of glucose is more likely mediated by GLUT1/4 and the activation of IGF1R and INSR.

### PI3K/AKT inhibition enhances MDL-800 anti-tumor efficacy in vitro and in vivo

**Fig. 4:**
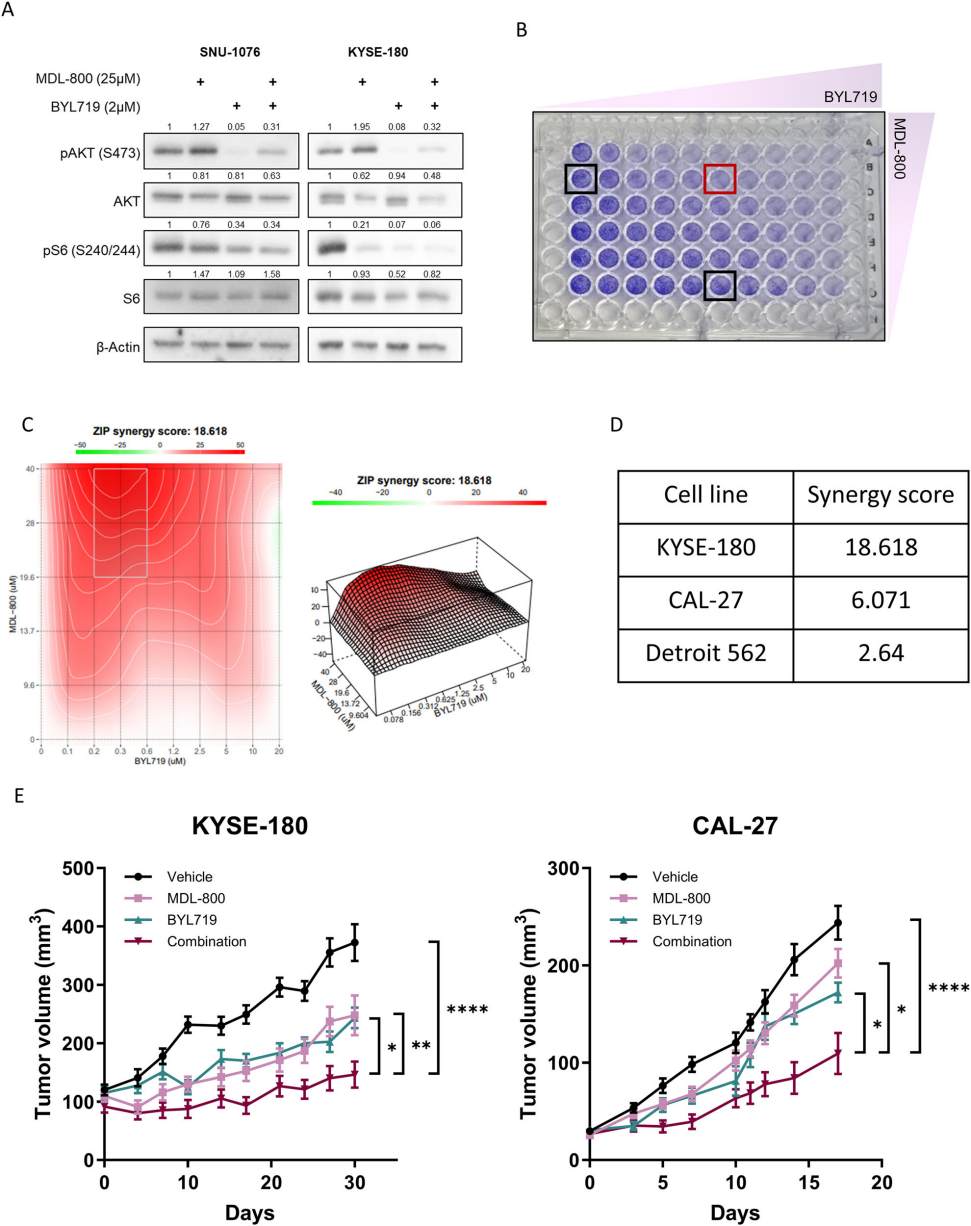
PI3K/AKT inhibition enhances MDL-800 anti-tumor efficacy in vitro and in vivo. **A** Western blot analysis for total protein and phosphorylated S6, and AKT in SNU-1076 and KYSE-180 cells treated with DMSO, 25 µM MDL-800 for 48 hours, 2 µM BYL719 for 24 hours, or both. β-Actin served as a loading control. Relative protein levels were calculated relative to β-Actin loading control and presented as fold change from the control sample. The data represent a representative experiment from three independent experiments. **B** Proliferation of KYSE-180 cells treated with increasing concentrations of both MDL-800 and BYL719 for 72 hours. Cells were fixed and stained with crystal violet. *N* = 3. **C** 2D & 3D synergy matrix showing the synergy scores of all MDL-800 and BYL719 concentration combinations in KYSE-180 cells treated as described in (**B**). **D** Synergy scores of different cell lines calculated as described in (**B**). **E** Tumor growth kinetics of KYSE-180 (Left Panel) and CAL-27 (Right Panel)-derived tumors in NSG mice (*n* = 5) treated with vehicle, MDL-800 (80 mg/kg), BYL719 (25 mg/kg), or both. Error bars indicate SE. Statistical significance was calculated by CGGC permutation test (*p < 0.05, **p < 0.01, ***p < 0.001, ****p < 0.0001).

To study whether inhibiting AKT via PI3K inhibition will sensitize cells to MDL-800 treatment, we decided to use the isoform-specific PI3Kα inhibitor BYL719, which has shown anti-proliferative activity and block PI3K/AKT/mTOR signaling in HNSCC and ESCC [33]. Western blot analysis showed that while MDL-800 increased pAKT and BYL719 reduced pAKT, the combined treatment of MDL-800 with BYL719 attenuated the AKT activation observed following MDL-800 treatment (Fig. 4A). This attenuation did not inhibit pAKT completely, indicating that the AKT pathway may be activated in a p110a-independent manner. To evaluate the efficacy of the combination of MDL-800 with BYL719, we tested the proliferation of cells treated with increasing concentrations of both drugs for 72 hours. By performing synergy analysis, we found that the combination of MDL-800 with BYL719 has a synergistic anti-proliferative effect in ESCC and HNSCC and cell lines (Fig. 4B–D and Supplementary Fig. 4A).

In light of the synergistic effect observed in vitro, we sought to test the efficacy of the combination of MDL-800 and BYL719 in vivo. To this end, we injected KYSE-180 and CAL-27 cells into NSG mice and treated them with vehicle, MDL-800 (80 mg/kg/2 days) and BYL719 (25 mg/kg/day), either separately or combined. Both MDL-800 and BYL719 induced growth delay of KYSE-180 tumors compared to the vehicle-treated group, though tumor progression was detected early on despite continuous treatments. However, the combination of MDL-800 and BYL719 showed a superior anti-tumor activity, with almost no detectable increase in KYSE-180-derived tumor volume, until 21 days of treatment (Fig. 4E). In line with the lower tumor volume, tumor weights at the end of the experiment of the combination group were lower compared to the single drug-treated groups (Supplementary Fig. 4B and C). In addition, reduction in KI67 staining was also observed (Supplementary Fig. 4D). Overall, these results indicate that blocking PI3K/AKT with BYL719 enhances the efficacy of MDL-800 in vitro and in vivo.

## Discussion

**Scheme 1:**
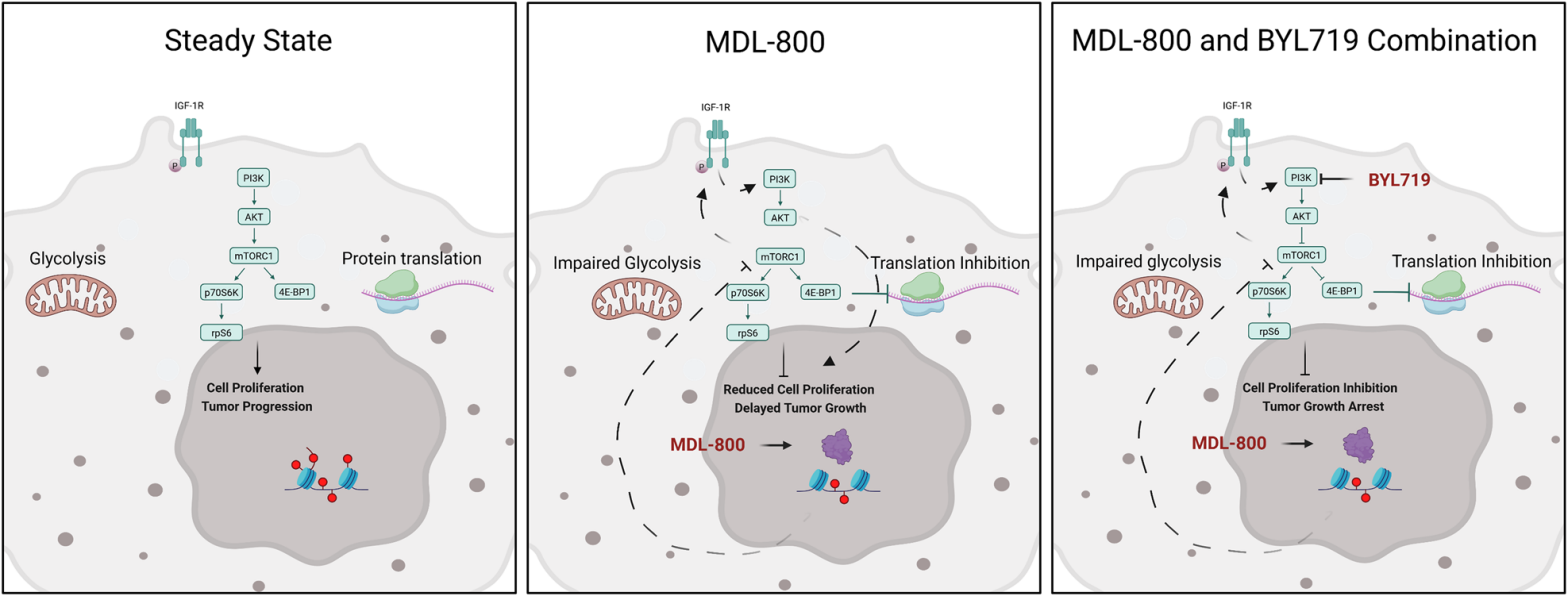
Left panel: A steady state is characterized by low SIRT6 expression and active PI3K/AKT/mTOR signaling that promotes glycolysis, protein translation, cell growth and survival. Middle panel: MDL-800 activates SIRT6, leading to the inhibition of mTOR, consequently inhibiting glycolysis, protein translation and cell proliferation. MDL-800 activity is attenuated by an upstream feedback loop activating AKT via IGF-1R/IR. Right panel: Blocking PI3K using BYL719 sensitizes cells to MDL-800 treatment and shows superior and synergistic anti-tumor activity. Created in BioRender. Elkabets, M. (2025)/o1ku47s.

In this study, we show for the first time that pharmacological activation of SIRT6 using the small-molecule MDL-800 is a potential therapeutic for HNSCC and ESCC. Treatment with MDL-800 showed anti-tumor activity in HNSCC and ESCC cell lines in vitro and in vivo, inhibiting tumor cell proliferation and migration. Mechanistically, MDL-800 inhibited mTOR activity and downstream signaling and consequently protein translation. mTOR inhibition was accompanied by an upstream feedback-loop activation of AKT, which was mediated by IGF-1R/IR activity. Finally, we identified that blocking PI3K using BYL719 sensitized cells to MDL-800 treatment and showed superior and synergistic anti-tumor activity (Scheme 1).

As most of the research addressing the role of SIRT6 in cancer is based on genetic models, there is still a limited amount of information regarding the therapeutic potential of SIRT6 activators. The first synthetic SIRT6 activator UBCS039 was shown to induce autophagy-related cell death in multiple cancer cell lines, including colon and non-small cell lung cancer, identifying the modulation of SIRT6 activity as a therapeutic strategy [34]. Recently, the small-molecule allosteric SIRT6 activator MDL-800 was shown to have anti-tumor activity in hepatocellular carcinoma, by promoting the deacetylation of H3K9ac and H3K56ac and inducing cell cycle arrest, consequently inhibiting cell proliferation, with IC_50_ values ranging between 18.6 μM to 24 μM [21]. Similarly, in non-small cell lung cancer, MDL-800 treatment was demonstrated to inhibit cell proliferation by inducing cell cycle arrest, with IC_50_ values ranging between 21.5 μM and 34.5 μM [22]. As part of its anti-tumor activity in HNSCC and ESCC, we demonstrated that MDL-800 treatment strongly inhibited cell proliferation in vitro and in vivo, with IC_50_ values ranging between 19.71 μM to 43.45 μM, similarly to recent publications (Fig. 1A).

In this work, we shed light on the mechanism by which pharmacological activation of SIRT6 using MDL-800 exerts its anti-tumor activity. Upon activation of SIRT6, we observed a substantial inhibition of cell proliferation, concomitant with the inhibition of glycolysis (Figs. 1 and 3C). This finding aligns with multiple previous studies that have demonstrated SIRT6 role as a negative regulator of glycolysis [18, 24]. The regulation of glycolysis by SIRT6 was shown to occur via the deacetylation of H3K9, and through co-repression of HIF-1α, ultimately resulting in reduced transcription of multiple glycolytic genes [24]. Consistently, we observed a downregulation of HIF-1α following treatment with MDL-800 (Fig. 2D and Supplementary Fig. 2C). Together, these results strongly indicate that SIRT6 activation with MDL-800 leads to the inhibition of glycolysis, possibly through the regulation of HIF-1α. Furthermore, we demonstrated that MDL-800 treatment inhibited mTOR activity in HNSCC and ESCC cell lines, resulting in the inhibition of S6 ribosomal protein and de-phosphorylation of 4E-BP1 (Fig. 3B). Additionally, MDL-800 treatment led to the inhibition of protein translation, a crucial process for cancer cell proliferation (Fig. 3A). These findings hold significance, as activating mutations in the PI3K/mTOR pathway, which is frequently altered in HNSCC and ESCC, play a pivotal role in driving cancer cell proliferation and promoting its survival [35, 36], and consequently have become attractive targets for the development of specific inhibitors [35, 37]. Taken together, our results highlight the therapeutic potential of MDL-800 and demonstrate its anti-tumor activity is mediated by the inhibition of oncogenic processes necessary for cancer progression and proliferation such as glycolysis and PI3K/mTOR signaling.

Multiple evidence has demonstrated that cancer cells develop escape or resistance mechanisms in response to treatment [10, 38], with recent publications implicating SIRT6 as a modulator of resistance for several therapies [39–41]. Thus, cancer cells may develop different mechanisms of cellular response to the pharmacological modulation of SIRT6. Here, we show that alongside the substantial suppression of mTOR activity, we observed an activation of AKT following MDL-800 treatment (Fig. 3B). Over recent decades, it was demonstrated that pharmacological inhibition of mTOR results in increased AKT phosphorylation and following activation [31, 42, 43]. Mechanistically, it was demonstrated that inhibition of mTOR relieves an mTORC1-induced inhibition of insulin and IGF-1 receptors, resulting in their re-activation and consequent AKT activation [31]. Notably, we observed increased activation of IGF-1R following MDL-800 treatment (Fig. 3D). Thus, we hypothesized that similarly, by inhibiting mTOR, MDL-800 treatment prevents the mTORC1-induced inhibition of IGF-1R, which results in IGF-1R activation. Furthermore, we speculated that the elevated activity of IGF-1R contributed to the observed activation of AKT following MDL-800 treatment. Markedly, combined treatment of MDL-800 with AEW541, an inhibitor targeting IGF-1R/IR, attenuated the MDL-800-induced activation of AKT (Fig. 3E). Additionally, as the insulin/IGF-1 signaling pathway plays a critical role in regulating cellular glucose uptake [44], it is possible that the observed activation of IGF-1R/IR may explain the increase in glucose uptake following MDL-800 treatment (Fig. 2F). However, further experimentation is needed to test this hypothesis. Taken together, these results suggest that the MDL-800-induced mTOR inhibition induces a feedback-loop activating IGF-1R, leading to AKT activation, and consequently limiting the anti-tumor activity MDL-800.

Considering the feedback-loop activating AKT following MDL-800 treatment, limiting its efficacy, we hypothesized that combining MDL-800 with the inhibition of PI3K/AKT using BYL719 will sensitize the cells to MDL-800 treatment. Indeed, our results demonstrated a superior and synergistic anti-tumor activity in vitro and in vivo following combination treatment of MDL-800 and BYL719 (Fig. 4). These findings strengthen our notion that the activation of AKT serves as a molecular compensatory mechanism employed by cells in response to SIRT6 activation.

Our study demonstrates the tumor-suppressive role of SIRT6 in ESCC. Presently, there is a limited amount of information available describing the role of SIRT6 in ESCC, and most of the existing data suggests an oncogenic role. Additionally, our work sheds further light on the role of SIRT6 in HNSCC. We comprehensively characterized the anti-tumor effect of SIRT6 in HNSCC and ESCC and showed it is mediated by inhibition of mTOR signaling and the modulation of cellular glucose metabolism. Moreover, we discovered SIRT6 activation triggers a compensatory feedback loop resulting in AKT activation and demonstrated that combining PI3K/AKT inhibition with SIRT6 activation sensitizes the cells and presents superior anti-tumor efficacy. Taken together, our findings highlight the role of SIRT6 as a tumor suppressor in HNSCC and ESCC and identify SIRT6 activation as a promising therapeutic strategy, either alone or in combination with PI3K inhibition.

### Limitations of the study

While we showed that SIRT6 activation leads to cell reprogramming, including reduced glycolysis, increased glucose uptake, and reduced protein synthesis, we did not show the direct role of SIRT6 on mTOR signaling which may explain the anti-tumor activity and the above-mentioned phenotypes [45]. Indicating that further investigation is required to explore how SIRT6 regulates mTOR under treatment with MDL-800. Although extensive biochemical profiling shows that MDL-800 is a potent and selective SIRT6 activator (with minor activity toward SIRT1), the definitive confirmation of off-target effects in our tumor models remains a limitation of this study and should be addressed in future work using orthogonal pharmacological tools—e.g., the structurally unrelated SIRT6 activators MDL-811 and UBCS039—to replicate the phenotype.

## Supplementary information

Supplementary Fig. 1

Supplementary Fig. 2

Supplementary Fig. 3

Supplementary Fig. 4

Supplementary Figure legends and Supplementary Materials and Methods

Original Data

Supplementary Table S1

Supplementary Table S2

Tumor volumes

## Data Availability

All data generated or analyzed during this study are included in this published article and its supplementary information files. The CUT&RUN datasets generated and analyzed during the current study are available in the NCBI repository, https://www.ncbi.nlm.nih.gov/sra/PRJNA1289082 (Accession ID: PRJNA1289082).

## References

[1] Johnson DE, Burtness B, Leemans CR, Lui VWY, Bauman JE, Grandis JR. Head and neck squamous cell carcinoma. Nat Rev Dis Primers 2020;6. 10.1038/s41572-020-00224-3. 10.1038/s41572-020-00224-3 PMC7944998 33243986

[2] Wang L, Pang W, Zhou K, Li L, Wang F, Cao W, et al. Characteristics of esophageal cancer in patients with head and neck squamous cell carcinoma. Transl Cancer Res. 2021;10:1954–61. 10.21037/tcr-20-2880. 35116518 10.21037/tcr-20-2880 PMC8799255

[3] Zhang H, Li H, Ma Q, Yang FY, Diao TY. Predicting malignant transformation of esophageal squamous cell lesions by combined biomarkers in an endoscopic screening program. World J Gastroenterol. 2016;22:8770–8. 10.3748/wjg.v22.i39.8770. 27818592 10.3748/wjg.v22.i39.8770 PMC5075551

[4] Pennathur A, Gibson MK, Jobe BA, Luketich JD. Oesophageal carcinoma. The Lancet, vol. 381, 2013. 10.1016/S0140-6736(12)60643-6. 10.1016/S0140-6736(12)60643-6 23374478

[5] Leemans CR, Snijders PJF, Brakenhoff RH. The molecular landscape of head and neck cancer. Nat Rev Cancer. 2018;18:269–82. 10.1038/nrc.2018.11. 29497144 10.1038/nrc.2018.11

[6] Yan W, Wistuba II, Emmert-Buck MR, Erickson HS. Squamous cell carcinoma - Similarities and differences among anatomical sites. Am J Cancer Res 2011;1:275–300. PMC3175764 21938273

[7] Ferlay J, Soerjomataram I, Dikshit R, Eser S, Mathers C, Rebelo M, et al. Cancer incidence and mortality worldwide: Sources, methods and major patterns in GLOBOCAN 2012. Int J Cancer. 2015;136:E359–86. 10.1002/ijc.29210. 25220842 10.1002/ijc.29210

[8] He S, Xu J, Liu X, Zhen Y. Advances and challenges in the treatment of esophageal cancer. Acta Pharm Sin B. 2021;11:3379–92. 10.1016/j.apsb.2021.03.008. 34900524 10.1016/j.apsb.2021.03.008 PMC8642427

[9] Li Q, Tie Y, Alu A, Ma X, Shi H. Targeted therapy for head and neck cancer: signaling pathways and clinical studies. Signal Transduct Target Ther 2023;8. 10.1038/s41392-022-01297-0. 10.1038/s41392-022-01297-0 PMC9842704 36646686

[10] Liu YP, Zheng CC, Huang YN, He ML, Xu WW, Li B. Molecular mechanisms of chemo- and radiotherapy resistance and the potential implications for cancer treatment. MedComm 2021;2. 10.1002/mco2.55. 10.1002/mco2.55 PMC8554658 34766149

[11] Byeon HK, Ku M, Yang J. Beyond EGFR inhibition: multilateral combat strategies to stop the progression of head and neck cancer. Exp Mol Med. 2019;51:1–14. 10.1038/s12276-018-0202-2. 30700700 10.1038/s12276-018-0202-2 PMC6353966

[12] Li Y, Jin J, Wang Y. SIRT6 widely regulates aging, immunity, and cancer. Front Oncol 2022;12. 10.3389/fonc.2022.861334. 10.3389/fonc.2022.861334 PMC9019339 35463332

[13] Raj S, Dsouza LA, Singh SP, Kanwal A. Sirt6 Deacetylase: A potential key regulator in the prevention of obesity, diabetes and neurodegenerative disease. Front Pharmacol. 2020;11. 10.3389/fphar.2020.598326. 10.3389/fphar.2020.598326 PMC7797778 33442387

[14] Onn L, Portillo M, Ilic S, Cleitman G, Stein D, Kaluski S, et al. SIRT6 is a DNA double-strand break sensor. Elife 2020;9. 10.7554/eLife.51636. 10.7554/eLife.51636 PMC7051178 31995034

[15] Chang AR, Ferrer CM, Mostoslavsky R. SIRT6, a mammalian deacylase with multitasking abilities. Physiol Rev 2020;100. 10.1152/physrev.00030.2018. 10.1152/physrev.00030.2018 PMC7002868 31437090

[16] Shen H, Qi X, Hu Y, Wang Y, Zhang J, Liu Z, et al. Targeting sirtuins for cancer therapy: epigenetics modifications and beyond. Theranostics. 2024;14:6726–67. 10.7150/thno.100667. 39479446 10.7150/thno.100667 PMC11519805

[17] Fiorentino F, Carafa V, Favale G, Altucci L, Mai A, Rotili D. The two-faced role of sirt6 in cancer. Cancers 2021;13. 10.3390/cancers13051156. 10.3390/cancers13051156 PMC7962659 33800266

[18] Choi JE, Sebastian C, Ferrer CM, Lewis CA, Sade-Feldman M, LaSalle T, et al. A unique subset of glycolytic tumour-propagating cells drives squamous cell carcinoma. Nat Metab 2021;3. 10.1038/s42255-021-00350-6. 10.1038/s42255-021-00350-6 PMC7954080 33619381

[19] Lai CC, Lin PM, Lin SF, Hsu CH, Lin HC, Hu ML, et al. Altered expression of SIRT gene family in head and neck squamous cell carcinoma. Tumor Biol 2013;34. 10.1007/s13277-013-0726-y. 10.1007/s13277-013-0726-y 23475622

[20] Wu X, Wang S, Zhao X, Lai S, Yuan Z, Zhan Y, et al. Clinicopathological and prognostic value of SIRT6 in patients with solid tumors: a meta-analysis and TCGA data review. Cancer Cell Int 2022;22. 10.1186/s12935-022-02511-3. 10.1186/s12935-022-02511-3 PMC8848894 35172823

[21] Huang Z, Zhao J, Deng W, Chen Y, Shang J, Song K, et al. Identification of a cellularly active SIRT6 allosteric activator. Nat Chem Biol. 2018;14. 10.1038/s41589-018-0150-0. 10.1038/s41589-018-0150-0 30374165

[22] Shang JL, Ning SB, Chen YY, Chen TX, Zhang J. MDL-800, an allosteric activator of SIRT6, suppresses proliferation and enhances EGFR-TKIs therapy in non-small cell lung cancer. Acta Pharm Sin. 2021;42:120–31. 10.1038/s41401-020-0442-2. 10.1038/s41401-020-0442-2 PMC7921659 32541922

[23] Elso CM, Roberts LJ, Smyth GK, Thomson RJ, Baldwin TM, Foote SJ, et al. Leishmaniasis host response loci (lmr1-3) modify disease severity through a Th1/Th2-independent pathway. Genes Immun. 2004;5:93–100. 10.1038/sj.gene.6364042. 14668789 10.1038/sj.gene.6364042

[24] Zhong L, D’Urso A, Toiber D, Sebastian C, Henry RE, Vadysirisack DD, et al. The Histone Deacetylase Sirt6 Regulates Glucose Homeostasis via Hif1α. Cell 2010;140. 10.1016/j.cell.2009.12.041. 10.1016/j.cell.2009.12.041 PMC2821045 20141841

[25] Chen EY, Tan CM, Kou Y, Duan Q, Wang Z, Meirelles GV, et al. Enrichr: Interactive and collaborative HTML5 gene list enrichment analysis tool. BMC Bioinformatics 2013;14. 10.1186/1471-2105-14-128. 10.1186/1471-2105-14-128 PMC3637064 23586463

[26] Sengupta S, Peterson TR, Sabatini DM. Regulation of the mTOR Complex 1 pathway by nutrients, growth factors, and stress. Mol Cell 2010;40. 10.1016/j.molcel.2010.09.026. 10.1016/j.molcel.2010.09.026 PMC2993060 20965424

[27] Ravi V, Jain A, Khan D, Ahamed F, Mishra S, Giri M, et al. SIRT6 transcriptionally regulates global protein synthesis through transcription factor Sp1 independent of its deacetylase activity. Nucleic Acids Res 2019;47. 10.1093/nar/gkz648. 10.1093/nar/gkz648 PMC6755095 31372634

[28] Toiber D, Stein D, Portillo M, Kopatch SK-, Stein D, Lachberg Y, et al. SIRT6 regulates protein synthesis and folding through nucleolar remodeling 2024. 10.21203/rs.3.rs-4215918/v1. 10.1111/acel.70384 PMC12913217 41703428

[29] Jefferies HBJ, Fumagalli S, Dennis PB, Reinhard C, Pearson RB, Thomas G. Rapamycin suppresses 5’TOP mRNA translation through inhibition of p70(s6k). EMBO J 1997;16. 10.1093/emboj/16.12.3693. 10.1093/emboj/16.12.3693 PMC1169993 9218810

[30] Qin X, Jiang B, Zhang Y. 4E-BP1, a multifactor regulated multifunctional protein. Cell Cycle. 2016;15:781–6. 10.1080/15384101.2016.1151581. 26901143 10.1080/15384101.2016.1151581 PMC4845917

[31] O’Reilly KE, Rojo F, She QB, Solit D, Mills GB, Smith D, et al. mTOR inhibition induces upstream receptor tyrosine kinase signaling and activates Akt. Cancer Res. 2006;66:1500–8. 10.1158/0008-5472.CAN-05-2925. 16452206 10.1158/0008-5472.CAN-05-2925 PMC3193604

[32] Gertman O, Omer D, Hendler A, Stein D, Onn L, Khukhin Y, et al. Directed evolution of SIRT6 for improved deacylation and glucose homeostasis maintenance. Sci Rep. 2018;8:3538. 10.1038/s41598-018-21887-9. 29476161 10.1038/s41598-018-21887-9 PMC5824787

[33] Wong CH, Ma BBY, Cheong HT, Hui CWC, Hui EP, Chan ATC. Preclinical evaluation of PI3K inhibitor BYL719 as a single agent and its synergism in combination with cisplatin or MEK inhibitor in nasopharyngeal carcinoma (NPC). Am J Cancer Res. 2015;5:1496–506. 26101713 PMC4473326

[34] Iachettini S, Trisciuoglio D, Rotili D, Lucidi A, Salvati E, Zizza P, et al. Pharmacological activation of SIRT6 triggers lethal autophagy in human cancer cells. Cell Death Dis. 2018;9:996. 10.1038/s41419-018-1065-0. 30250025 10.1038/s41419-018-1065-0 PMC6155207

[35] Marquard FE, Jücker M. PI3K/AKT/mTOR signaling as a molecular target in head and neck cancer. Biochem Pharm. 2020;172:113729. 10.1016/j.bcp.2019.113729. 31785230 10.1016/j.bcp.2019.113729

[36] Luo Q, Du R, Liu W, Huang G, Dong Z, Li X PI3K/Akt/mTOR Signaling Pathway: Role in Esophageal Squamous Cell Carcinoma, Regulatory Mechanisms and Opportunities for Targeted Therapy. Front Oncol 2022;12. 10.3389/fonc.2022.852383. 10.3389/fonc.2022.852383 PMC8980269 35392233

[37] Huang R, Dai Q, Yang R, Duan Y, Zhao Q, Haybaeck J, et al. A Review: PI3K/AKT/mTOR Signaling Pathway and Its Regulated Eukaryotic Translation Initiation Factors May Be a Potential Therapeutic Target in Esophageal Squamous Cell Carcinoma. Front Oncol 2022;12. 10.3389/fonc.2022.817916. 10.3389/fonc.2022.817916 PMC9096244 35574327

[38] Lackner MR, Wilson TR, Settleman J. Mechanisms of acquired resistance to targeted cancer therapies. Future Oncol. 2012;8:999–1014. 10.2217/fon.12.86. 22894672 10.2217/fon.12.86

[39] Khongkow M, Olmos Y, Gong C, Gomesl AR, Monteiro LJ, Yagüe E, et al. SIRT6 modulates paclitaxel and epirubicin resistance and survival in breast cancer. Carcinogenesis. 2013;34:1476–86. 10.1093/carcin/bgt098. 23514751 10.1093/carcin/bgt098

[40] Strub T, Ghiraldini FG, Carcamo S, Li M, Wroblewska A, Singh R, et al. SIRT6 haploinsufficiency induces BRAFV600E melanoma cell resistance to MAPK inhibitors via IGF signalling. Nat Commun. 2018;9:3440. 10.1038/s41467-018-05966-z. 30143629 10.1038/s41467-018-05966-z PMC6109055

[41] Yang J, Li Y, Zhang Y, Fang X, Chen N, Zhou X, et al. Sirt6 promotes tumorigenesis and drug resistance of diffuse large B-cell lymphoma by mediating PI3K/Akt signaling. J Exp Clin Cancer Res. 2020;39:142 10.1186/s13046-020-01623-w. 32711549 10.1186/s13046-020-01623-w PMC7382040

[42] Zou Z, Tao T, Li H, Zhu X. MTOR signaling pathway and mTOR inhibitors in cancer: Progress and challenges. Cell Biosci. 2020;10:31 10.1186/s13578-020-00396-1. 32175074 10.1186/s13578-020-00396-1 PMC7063815

[43] Bergholz JS, Zhao JJ. How compensatory mechanisms and adaptive rewiring have shaped our understanding of therapeutic resistance in cancer. Cancer Res. 2021;81:6074–7. 10.1158/0008-5472.CAN-21-3605. 34911779 10.1158/0008-5472.CAN-21-3605 PMC9033251

[44] Kasprzak A. Insulin-like growth factor 1 (Igf-1) signaling in glucose metabolism in colorectal cancer. Int J Mol Sci. 2021;22:6434 10.3390/ijms22126434. 34208601 10.3390/ijms22126434 PMC8234711

[45] Glaviano A, Foo ASC, Lam HY, Yap KCH, Jacot W, Jones RH, et al. PI3K/AKT/mTOR signaling transduction pathway and targeted therapies in cancer. Mol Cancer. 2023;22:138. 10.1186/s12943-023-01827-6. 37596643 10.1186/s12943-023-01827-6 PMC10436543

