# Supplementary material for: Pharmacological activation of SIRT6 suppresses progression of head and neck and esophageal squamous cell carcinoma by modulation of cellular metabolism and protein translation
Source: Cell Death Dis. 2025 Oct 16;16(1):727. doi: 10.1038/s41419-025-07959-5 (PMC12533074; doi:10.1038/s41419-025-07959-5)
Supplement: Supplementary file 5 — Supplementary Figure legends and Supplementary Materials and Methods [file 41419_2025_7959_MOESM5_ESM.docx]

**Supplementary Figure legends:**

**Supplementary Figure 1**

KYSE180 cells were treated with DMSO, 25uM or 50uM of MDL-800. 48 hours post-treatment: **(A)** Dose-response curve of the indicated cell lines following 72 hours of treatment with increasing concentrations of MDL-800 (0-100uM), N=3. **(B)** Cell cycle analysis was performed, and the fraction of the cells in each phase was determined by flow cytometry. Error bars indicate SE, N=2. **(C)** Apoptosis assay was performed by flow cytometry using staining with annexin V-FITC and propidium iodide. Error bars indicate SE, N=3. **(D)** Tumor growth kinetics of HSC-2 (Left panel) and FaDu (Right panel) derived tumors in NSG mice (n=4) treated with vehicle or MDL-800 (80 mg/kg). Error bars indicate SE. Statistical significance was calculated by CGGC permutation test (*p < 0.05, ***p* < 0.01, ****p* < 0.001, *****p* < 0.0001). **(E)** KI67 IHC staining of FaDu-derived tumors from vehicle and MDL-800 treated mice. The percentage of positive cells was calculated and presented in a bar graph. Error bars indicate SE. Statistical significance was calculated using unpaired t-test (*p < 0.05, **p < 0.01, ***p < 0.001, ****p <0.0001).

**Supplementary Figure 2**

**(A)** Membranes of protein array blots of SNU-1076 and KYSE-180 cells treated with DMSO or 25µM MDL-800 for 48 hours. **(B)** Bar graph showing the 10 most enriched downregulated pathways sorted by p-value ranking in SNU-1076 and KYSE-180 cells following 48 hours MDL-800 treatment. **(C)** Western blot analysis for HIF-1α in SNU-1076 and KYSE-180 cells treated with DMSO or 25µM MDL-800 for 24 and 48 hours. β-Actin served as a loading control. Relative protein levels were calculated relative to β-Actin loading control and presented as fold change from the control sample. Data represents a representative experiment from two independent experiments.

**Supplementary figure 3**

**(A)** Western blot analysis for SUnSET assay in SNU-1076 and KYSE-180 cells treated with puromycin for 20 minutes following 24 hours treatment of 25µM MDL-800. Cells treated with 50µg/ml cycloheximide (CHX) for 1 hour served as a negative control. Membranes were stained with ponceau prior to immunoblotting and β-Actin served as a loading control. Data represents a representative experiment from two independent experiments. **(B)** Western blot analysis for total protein and phosphorylated AMPK, and ACC in SNU-1076 and KYSE-180 cells treated with DMSO or 25µM MDL-800 for 24 and 48 hours. β-Actin served as a loading control. Relative protein levels were calculated relative to β-Actin loading control and presented as fold change from the control sample. Data represents a representative experiment from three independent experiments.

**(C)** Membranes of RTK array blots of SNU-1076 and KYSE-180 cells treated with DMSO or 25µM MDL-800 for 48 hours.

**Supplementary figure 4**

**(A)** 2D & 3D synergy matrix showing the synergy scores of the MDL-800 and BYL719 in all concentration combinations in CAL-27 and Detroit-562 cells treated with increasing concentrations of both MDL-800 and BYL719 for 72 hours. n=3. (**B**) Tumor weighs of KYSE-180 (Left Panel) and CAL-27 (Right Panel)-derived tumors in NSG mice treated with vehicle, MDL-800 (80 mg/kg) BYL719 (25 mg/kg) or both. Tumor weights and means were calculated and presented in a bar graph. Error bars indicate SE. Statistical significance was calculated using one-way ANOVA with Tukey’s multiple comparison test (*p < 0.05, **p < 0.01, ***p < 0.001, ****p <0.0001). (**C**) CAL-27-derived tumors collected at the termination of the experiment described in **B. (D)** KI67 IHC staining of CAL-27-derived tumors from vehicle, MDL-800, BYL719 and combination treated mice. The percentage of positive cells was calculated and presented in a bar graph. Error bars indicate SE. Statistical significance was calculated using unpaired t-test (*p < 0.05, **p < 0.01, ***p < 0.001, ****p <0.0001).

**Supplementary Materials and Methods**

**Cell Lines and Chemical Compounds**

The human HNSCC cell lines SNU-1076 (KCLB), CAL33 (DSMZ), HSC-2(HSRRB), CAL27, Detroit562 and FaDu (ATCC) and ESCC cell line KYSE-180(DSMZ), were purchased from the mentioned commercial vendors. All cell lines were maintained at 37° in a humidified atmosphere at 5% CO2 in Dulbecco’s Modified Eagle’s medium (DMEM) or RPMI-1640 medium supplemented with 1% L-glutamine 200mM, 100 units of penicillin and streptomycin 10% fetal bovine serum (FBS).

**Antibodies and reagents**

The following antibodies were purchased from Cell Signaling Technology: anti-pAKT Ser473 (#4060), anti-AKT (#4691), anti-pS6 S235/236 (#4858), anti-pS6 S240/244 (#5364), anti-S6 ribosomal protein (#2217), anti-β-Actin (#4970), anti-p4E-BP1 Thr37/46 (#2855), anti-4E-BP1 (#9644), anti-HIF-1α (#36169), anti-SIR6 (#12486), anti- Acetyl-Histone H3 (Lys56) (#4243), anti-pAMPKα T172 (#2535), anti-ACC2 (#8578), anti-pACC2 S79 (#3661). Anti- Histone H3K9ac (39317) was purchased from Active Motif, anti-Histone H3 (ab1791) and anti-AMPKα (SAB1412357) from Abcam and Anti-Puromycin (MABE343) from Sigma-Aldrich. MDL-800 for in vitro experiments was purchased from MedChemExpress (HY-119376) and Sigma-Aldrich (SML2529). MDL-800 for In vivo experiments was kindly provided by Prof. Jian Zhang. BYL719 and AEW541 were provided by Novartis Pharma AG (Basil, Switzerland). All compounds were dissolved in dimethyl sulfoxide (DMSO) for in vitro uses and in 5% DMSO (D8418, Sigma-Aldrich), 30% PEG-300(202371, Sigma-Aldrich), and 65% phosphate-buffered saline (PBS) (02-023-1A, SARTORIUS) or 0.5% carboxymethylcellulose (CMC) (9481, Sigma-Aldrich) for in vivo uses.

**IC50, synergy, and proliferation assays**

Cells were seeded in a 96-well plate (5K-7K cells/well), treated with increasing concentrations of MDL-800 (0-100μM) or MDL-800 (0-40μM) and BYL719 (0-20μM and allowed proliferation for 72 hours. Then, cells were stained with crystal violet (1g/L) for 5 minutes, rinsed and dried. Cell-bound crystal violet was dissolved with 10% acetic acid, and absorbance was measured at 570 nm (BioTek™ Epoch™ spectrophotometer). IC50 values calculations and dose-response curves were plotted using GraphPadPrism 9 software. For synergy assay, the colorimetric results were analyzed, and ZIP Synergy scores were calculated using the Synergyfinder website (https://synergyfinder.fimm.fi)[1].

For cell proliferation assay, cells were seeded in a 24-well plate (30K-60K cells/well) and treated with DMSO, 25μM or 50μM MDL-800 the following day. Live cell imaging and time series recording to record cell confluence every 6 hours were performed using the JuliTM Stage Real-Time Cell History Recorder. After 72 hours, cells were stained and quantified as in the IC50 assay. Cell proliferation was determined as the relative percentage of control DMSO-treated cells, and the percentage of growth inhibition was calculated.

**Apoptosis and Cell Cycle Analysis**

Cells were seeded in a 6-well plate (500K cells/well) and treated with DMSO, 25μM or 50μM MDL-800 for 48 hours. Cells and supernatant were collected into 15-mL tubes and centrifuged for 10 min at 4 °C. The pellet was washed twice with ice-cold PBS and stained in the dark with 50μL of propidium iodide staining solution (6990-42, eBioscience) and Annexin V FITC (11-8005-74, eBioscience) for 20 min at room temperature. For cell cycle analysis, the cell pellet was fixed at −20 °C with 70% ethanol and stored for at least 24 hours at −20 °C. After fixation, the cell pellet was washed twice with ice-cold PBS, treated with 50 μL of RNase solution (100 μg/μL) for 30 min at 37 °C, and stained in the dark with 50μL of propidium iodide solution (100 μg/mL, Sigma-Aldrich) for 30 min at room temperature. The apoptotic cell population and cell cycle phase were analyzed using FlowJo software v10.8.1.

**Migration assay**

Cells were seeded in a 6-well plate (500K cells/well) and treated with DMSO or 25μM for 24 hours. After 24 hours, cells were suspended in serum-free RPMI, and 120K cells were seeded in the upper chamber of a ThinCert® insert (662638, Grainer), which was placed in a 24-well plate containing 10% FBS-supplemented RPMI. After another 24 hours, the insert membranes were fixed and stained using DIF-STAIN KIT (1526, KALTEK), mounted on slides, and scanned. The number of migrated cells was quantified using the Qupath-0.2.3 software.

**In vivo experiments**

NOD.Cg-Prkdc^scid^ Il2rg^tm1Wjl^/SzJ (NOD SCID gamma; NSG) mice were purchased from The Jackson Laboratory. Mice were housed in air-filtered laminar flow cabinets with a 12/12-hour light/dark cycle and were fed food and water ad libitum. Mice were maintained and treated according to the institutional guidelines of Ben-Gurion University of the Negev, and the experiments were approved by the Institutional Animal Care and Use Committee [IL-57-12-2022E]. To generate cell-derived xenografts, KYSE-180 and HSC-2 cells (10 × 106 and 5 × 106 cells, respectively) were suspended in 60μl of PBS and injected subcutaneously into the backs of 6–8 weeks old female NSG mice. When tumors reached the volume of 100mm3, mice were randomized into 2 or 4 groups of 4-6 mice per group, depending on the experiment. In all In vivo experiments, MDL-800 (80mg/kg) was dissolved in 5% DMSO, 30% PEG300, and 65% PBS and administered every other day by intraperitoneal injection (IP), and BYL719 (25mg/kg) was dissolved in 5% CMC and administered daily by oral gavage. Drug-treated mice received MDL-800, BYL719, or both, depending on the experiment. Vehicle-treated mice received 5% DMSO, 30% PEG300, 65% PBS, 5% CMC, or both, depending on the experiment. Tumors were measured with a digital caliber twice a week, and tumor volumes were calculated according to the formula: Tumor volume = (L × W × W), where W stands for tumor width and L for tumor length. At the end of the experiments, animals were euthanized with CO2, and the tumors were harvested, fixed in 4% paraformaldehyde (PFA) overnight, and stored in 70% ethanol for investigation. Measurements of tumor volumes are plotted as means ± SEM.

**Immunohistochemistry**

After being maintained in 70% ethanol, PFA-fixed tumors were embedded in Paraffin, sectioned into 5-μm slices, loaded onto microscope slides, and deparaffinized at 60°C for 1 hour. After additional deparaffinization with a xylene substitute (Leica, 3803672E) and rehydration steps in a descending alcohol series, antigen retrieval was performed. The slides were incubated in 10 mM citric acid buffer, pH 6.0 at 100 °C for 15 min, then cooled at room temperature and rinsed with doubly distilled water (DDW) for 3 minutes, three times. Next, endogenous peroxidases were inactivated with 0.3% hydrogen peroxide in methanol buffer for 30 min. The slides were then washed three times with PBS for 3 minutes and blocked with 5% BSA in PBS-T (0.1% TWEEN) for 1 hour at room temperature. Slides were then incubated overnight at 4 °C with the primary antibodies, Ki67 (Sigma-Aldrich, AB9260), pS6 (CST, 4857S), and H3K9ac (Abcam, ab10812), diluted in blocking solution. The following day, the slides were washed three times with PBS-T. The VECTASTAIN®ABC kit (Vector Laboratories, VE-PK-6200) was used for antibody detection according to the manufacturer's protocol, with 3,3'-diaminobenzidine (DAB) (ACH500-IFU; ScyTek laboratories) as a substrate for color development. Lastly, slides were counterstained with hematoxylin, dehydrated, and covered using a mounting medium (Sub-X, Leica 3801740). All slides were digitalized using the Panoramic Scanner (3DHISTECH, Budapest, Hungary), and analysis was performed using the Qupath-0.2.3 software.

**Cleavage under targets & release using nuclease (CUT&RUN) assay**

Cells were seeded in a 100mm plate (2M cells/plate) and treated with DMSO, or 25μM MDL-800. After 48 hours, cells were harvested, resuspended in nuclear extraction buffer (20 mM 4-[2-hydroxyethyl]-1-piperazineethanesulfonic acid, pH = 7.9, 10 mM potassium chloride, 0.1% Triton X-100, 20% glycerol, 0.5 mM spermidine) supplemented with cOmpleteTM EDTA-free protease inhibitor cocktail (Roche) and kept on ice for 10 minutes. Then, nuclei were centrifuged at 600g for 3 minutes at 4 °C, resuspended in nuclear extraction buffer, and subjected to Cleavage Under Targets & Release Using Nuclease (CUT&RUN) using the CUTANA ChIC/CUT&RUN kit (EpiCypher). Next, nuclei were attached to concanavalin A beads, permeabilized with digitonin, and incubated with anti-Acetyl-Histone H3 (Lys9) (9649T, Cell signaling technology, 1:100) and anti-IgG (EpiCypher; 1:100) on nutator overnight at 4 °C. The next day, nuclei were washed twice with Digitonin buffer (EpiCypher) and incubated with pAG-MNase (EpiCypher) for 10 minutes at room temperature. Then, MNase was activated with 1mM calcium chloride for 2 hours at 4 °C. Stop buffer supplemented with E coli spike-in DNA (EpiCypher) was then added to stop the reaction. Library preparation was done according to a standard protocol using the CUTANA CUT&RUN Library Prep Kit (EpiCypher). Constructed CUT&RUN libraries were sequenced on an Illumina NovaSeq platform, obtaining 150-bp paired-end reads.

**Analysis of global H3K9ac acetylation under SIRT6 activation treatment**

Paired-end Fastq files from CUT&RUN analysis were processed as follows, using NeatSeq-Flow platform{https://doi.org/10.1101/173005}. Samples were sequenced at an average depth of 26M reads (range of 20 to 33M reads per sample). Reads were trimmed using TrimGalore v0.6.10 and CutAdapt v4.9 [2]. Reads were then aligned to the hg38 genome using bowtie2 v2.5.4 [3], and samtools v1.21 [4] was used for alignment filtering, sorting and conversion to the BAM format. Quality of reads and alignments was visualized with MultiQC [5].

BAM files were analyzed with Csaw v.1.38.0 [6] on R v.4.4.1. Mapped paired-end reads were filtered to include only reads shorter than 400bp and with quality score higher than 20 and were counted in sliding windows of width 50. Windows were filtered by comparison with the global background abundance computed from windows of 10Kbp, removing bins with < 3.5 log-fold-change (logFC) abundancy relative to the background. Normalization factors were calculated to remove efficiency biases. R package edgeR v. 4.2.2 [7] was used to estimate dispersions and find differential binding between samples, and limma package v.3.60.6 [8] was used to create an MDS plot using the 5000 most differential peaks (Figure 1b). DB peaks were clustered into regions surrounding (3000 kbp up- and downstream) known genes in the hg38 human genome. Each region was annotated by finding overlapping genes within 7Kbp of the regions. The region’s representative logFC values were used to rank the regions, from highest to lowest, where high logFC represents higher abundance in SIRT6 treated samples.

For additional QC, SEACR v. 1.3 [9] was used to call enriched peaks. Using these peaks, the *deeptools* package v. 3.5.4 [10] was applied to create a heatmap of the genome-wide peaks coverage (“Tornado plots”) 3Kbp up- and down-stream from the gene transcription start sites (TSS), as determined by hg38 GTF file.

The genes associated with enriched peaks, with differential representation of reads, when comparing SIRT6 treatment to control were determined by physical proximity. Thus, genes were ranked based on a summary statistic of their enrichment under SIRT6 treatment (logFC of peaks intensity). Next, we performed Gene Set Enrichment Analysis (GSEA) to establish the pathways that are significantly associated with SIRT6 treatment as reflected in the differential representation of peaks using *fgsea* Bioconductor package v. 1.30.0 {doi:10.1101/060012} with Gene Ontology Biological Process collection of gene sets and pathways [11]. Raw data available at: https://www.ncbi.nlm.nih.gov/sra/PRJNA1289082 (Accession ID: PRJNA1289082).

**Seahorse real-time metabolic analysis**

Cells were seeded (20K cells/well) in Seahorse XFe 96-well plate (Agilent, Cat # 101085-004) and treated with DMSO or 25μM MDL-800 for 24 or 48 hours. 24 hours before the experiment, cartridge plates were immersed in XF calibrant solution (Agilent, Cat #100840-000) and placed in a CO2-free incubator until used. On the day of the experiment, 2 hours before Oxygen Consumption Rate (OCR) and Extracellular Acidification Rate (ECAR) measurements, medium was replaced by Seahorse assay DMEM supplemented with 2mM Glutamine for Glycolysis Stress assay or with 2mM Glutamine (Sartorius, 03-020-1B), 10mM Glucose (GERBU, 2028) and 1mM Pyruvate (Sigma Aldrich, P2256) for Mitochondrial Stress assay, and plates were kept at 37°C without CO2. In the meantime, ports of the cartridge containing the probes were loaded with the compounds that will be injected during the assays and the cartridge was calibrated. For Glycolysis Stress assay Oligomycin was used at a final concentration of 1.5μM (Cayman, 11342), Glucose at a final concentration of 5 mM (GERBU, 2028), and 2-Deoxy-Glucose (2-DG; Santa Cruz Biotechnology, sc-202010) at final concentration of 50mM. For Mitochondrial Stress assay, Oligomycin was used at a final concentration of 1.5μM (Cayman, 11342), FCCP at a final concentration of 1.5μM (Sigma Aldrich, C2920), and antimycin (Sigma Aldrich, A8674) and Rotenone (Sigma Aldrich, 557368) were used at final concentrations of 0.5μM. The assay was performed using Seahorse Bioscience XF96 extracellular flux analyzer, and statistics were exported and analyzed using Seahorse Wave Desktop Software v2.6.3.5.

**Glucose uptake assay**

Cells were seeded in a 24-well plate (50K cells/well) and treated with DMSO, 25μM MDL-800 for 24 or 48 hours and kept in glucose-free RPMI. Three to four hours prior to the end of the treatment, cells were treated with 2-NBDG. Then, cells were collected, washed, and the assay was performed according to the manufacturer’s instructions (Cayman, Ann Arbor, MI, USA). The amount of fluorescent 2-NBDG in the cells was measured at 485/535nm and analyzed using FlowJo software v10.8.1.

**Proteome profiler arrays**

1.5 million Cells were seeded in 100mm plates and allowed to adhere overnight. Next, cells were then treated with 25μM MDL-800 or DMSO for 48 hours. Total cell lysates of 300μg were analyzed using the Proteome Profiler™ Human Phospho-RTK antibody array, catalog # ARY001B and Proteome Profiler™ Human XL Oncology Array catalog # ARY026 (R&D Systems, Burlington, ON, Canada), according to the manufacturer's protocol. The arrays were visualized, and images were captured by C300 Azure Biosystems camera system (Azure Biosystems). Densitometric analysis was performed using ImageJ software with the Protein Array Analyzer plugin[12]. Pathway enrichment analysis was performed using the online available tool Enrichr[13].

**Western Blotting**

Cells were washed with ice-cold PBS and scraped into ice-cold lysis buffer with phosphatase inhibitor cocktail (Stratech, B15001-BIT) and protease inhibitor (MilliporeSigma, P2714-1BTL). Lysates were centrifuged at 14,000 rpm for 10 min at 4°, and supernatants were collected and assayed for protein concentration using the Bradford assay (Bio-Rad, Protein Assay, cat# 500–0006). Twenty micrograms of total lysates were loaded to run SDS-PAGE and electrophoretically transferred onto a PVDF membrane (Bio-Rad, 1704157). Membranes were blocked for 1 hr in 5% BSA in Tris-buffered saline Tween (TBS-T) and then hybridized using primary antibodies in 5% BSA TBS-T. Rabbit horseradish peroxidase (HRP)-conjugated secondary antibodies (1:50,000, Jackson) were diluted in 5% BSA in TBS-T. Protein-antibody complexes were detected by chemiluminescence using Enhanced chemiluminescence (Westar Nova 2.0 Cyanagen XLS071.0250), and images were captured using C300 Azure Biosystems camera system (Azure Biosystems). Protein expression was quantified using IMAGEJ software. Relative protein levels were calculated relative to β-Actin loading control and presented as fold change from the control sample.

**SUnSET assay**

Cells were seeded in a 6-well plate and treated the next day with DMSO or 25μM MDL-800 for 48 hours. After 48 hours of treatment, the SUnSET assay[14] was performed by adding puromycin (Thermo Fisher Scientific, A1113803) at a final concentration of 10 μg/ml directly to the cell media for incubation of 20 minutes in 37°C. Cycloheximide (Sigma Aldrich, 01810) treated cells (0.5μM) were used as negative control. After puromycin labeling, cells were collected for total protein extraction and Western blot analysis.

**Statistical analysis**

Statistical analysis was performed using GraphPad Prism software version 9, and results are presented as means ± SEM. For comparisons between two groups, P values were calculated using unpaired t-test. For comparisons between three or more groups, P values were calculated using one-way ANOVA with Tukey’s multiple comparison test (*p < 0.05, **p < 0.01, ***p < 0.001). Statistical analysis for protein and RTK arrays was performed using unpaired multiple t-tests with false discovery rate (FDR) control, applying the two-stage step-up method by Benjamini, Krieger, and Yekutieli. Statistical analysis for comparing growth curves from in vivo experiments was performed using CGGC permutation tests developed by Walter& Elisa Hall bioinformatics - Institute of Medical Research - [http://bioinf.wehi.edu.au/software/compareCurves/index.html[14](http://bioinf.wehi.edu.au/software/compareCurves/index.html%5b14)[15].

**Bibliography**

[1] Ianevski A, Giri AK, Aittokallio T. SynergyFinder 3.0: an interactive analysis and consensus interpretation of multi-drug synergies across multiple samples. Nucleic Acids Res 2022;50:W739–43. https://doi.org/10.1093/nar/gkac382.

[2] Martin M. Cutadapt Removes Adapter Sequences from High-Throughput Sequencing Reads. EMBnet J 2011;17:10–2. https://doi.org/10.14806/ej.17.1.200.

[3] Langmead B, Salzberg SL. Fast gapped-read alignment with Bowtie 2. Nat Methods 2012;9:357–9. https://doi.org/10.1038/nmeth.1923.

[4] Li H, Handsaker B, Wysoker A, Fennell T, Ruan J, Homer N, et al. The Sequence Alignment/Map format and SAMtools. Bioinformatics 2009;25:2078–9. https://doi.org/10.1093/bioinformatics/btp352.

[5] Ewels P, Magnusson M, Lundin S, Käller M. MultiQC: Summarize analysis results for multiple tools and samples in a single report. Bioinformatics 2016;32:3047–8. https://doi.org/10.1093/bioinformatics/btw354.

[6] Lun ATL, Smyth GK. Csaw: A Bioconductor package for differential binding analysis of ChIP-seq data using sliding windows. Nucleic Acids Res 2015;44:e45. https://doi.org/10.1093/nar/gkv1191.

[7] Robinson MD, McCarthy DJ, Smyth GK. edgeR: A Bioconductor package for differential expression analysis of digital gene expression data. Bioinformatics 2009;26:139–40. https://doi.org/10.1093/bioinformatics/btp616.

[8] Ritchie ME, Phipson B, Wu D, Hu Y, Law CW, Shi W, et al. Limma powers differential expression analyses for RNA-sequencing and microarray studies. Nucleic Acids Res 2015;43:e47. https://doi.org/10.1093/nar/gkv007.

[9] Meers MP, Tenenbaum D, Henikoff S. Peak calling by Sparse Enrichment Analysis for CUT&RUN chromatin profiling. Epigenetics Chromatin 2019;12. https://doi.org/10.1186/s13072-019-0287-4.

[10] Ramírez F, Ryan DP, Grüning B, Bhardwaj V, Kilpert F, Richter AS, et al. deepTools2: a next generation web server for deep-sequencing data analysis. Nucleic Acids Res 2016;44:W160–5. https://doi.org/10.1093/NAR/GKW257.

[11] Ashburner M, Ball CA, Blake JA, Botstein D, Butler H, Cherry JM, et al. Gene ontology: Tool for the unification of biology. Nat Genet 2000;25:25–9. https://doi.org/10.1038/75556.

[12] Carpentier G. Protein Array Analyzer for ImageJ. ImageJ User and Developer Conference 2010.

[13] Chen EY, Tan CM, Kou Y, Duan Q, Wang Z, Meirelles G V., et al. Enrichr: Interactive and collaborative HTML5 gene list enrichment analysis tool. BMC Bioinformatics 2013;14. https://doi.org/10.1186/1471-2105-14-128.

[14] Schmidt EK, Clavarino G, Ceppi M, Pierre P. SUnSET, a nonradioactive method to monitor protein synthesis. Nat Methods 2009;6. https://doi.org/10.1038/nmeth.1314.

[15] Elso CM, Roberts LJ, Smyth GK, Thomson RJ, Baldwin TM, Foote SJ, et al. Leishmaniasis host response loci (lmr1-3) modify disease severity through a Th1/Th2-independent pathway. Genes Immun 2004;5:93–100. https://doi.org/10.1038/sj.gene.6364042.
