# Supplementary material for: Pharmacological activation of SIRT6 suppresses progression of head and neck and esophageal squamous cell carcinoma by modulation of cellular metabolism and protein translation
Source: Cell Death Dis. 2025 Oct 16;16(1):727. doi: 10.1038/s41419-025-07959-5 (PMC12533074; doi:10.1038/s41419-025-07959-5)

Figure 1B

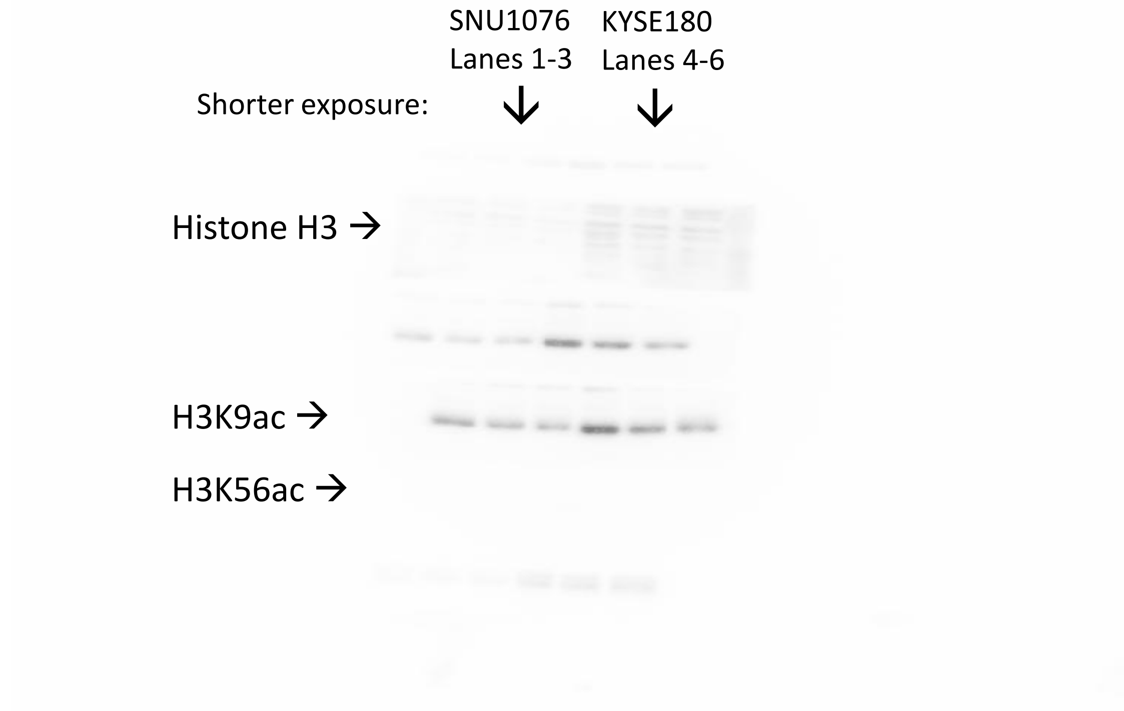
SNU1076
Lanes 1-3
KYSE180
Lanes 4-6
Shorter exposure:
Histone H3 →
H3K9ac →
H3K56ac →

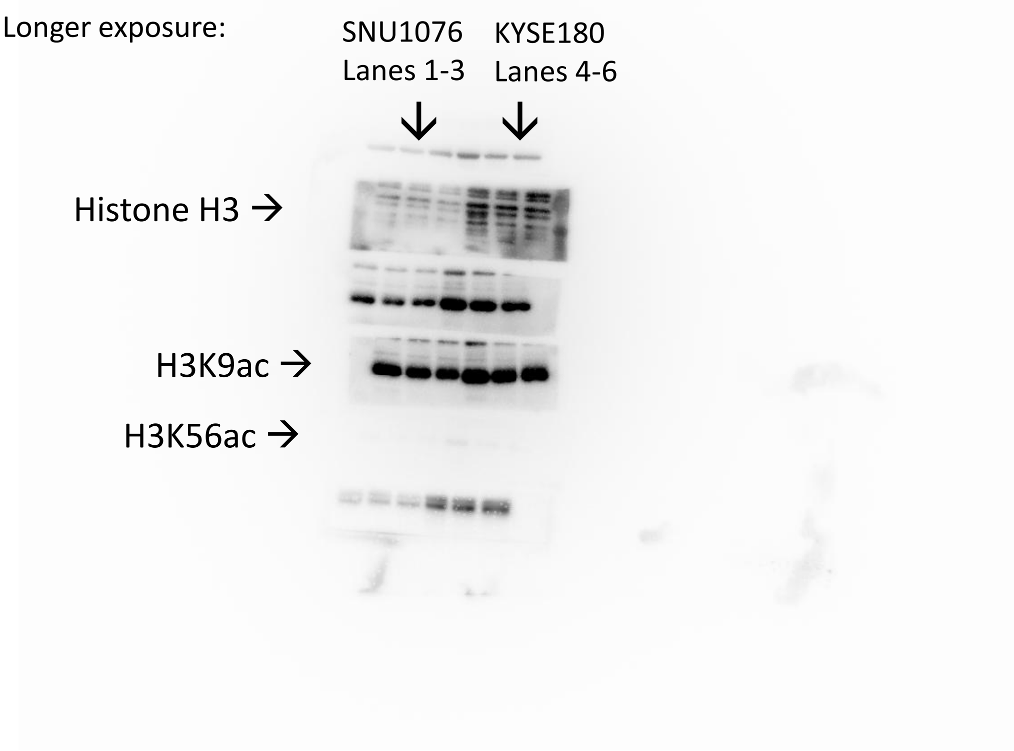
Longer exposure:
SNU1076
Lanes 1-3
KYSE180
Lanes 4-6
Histone H3 →
H3K9ac →
H3K56ac →

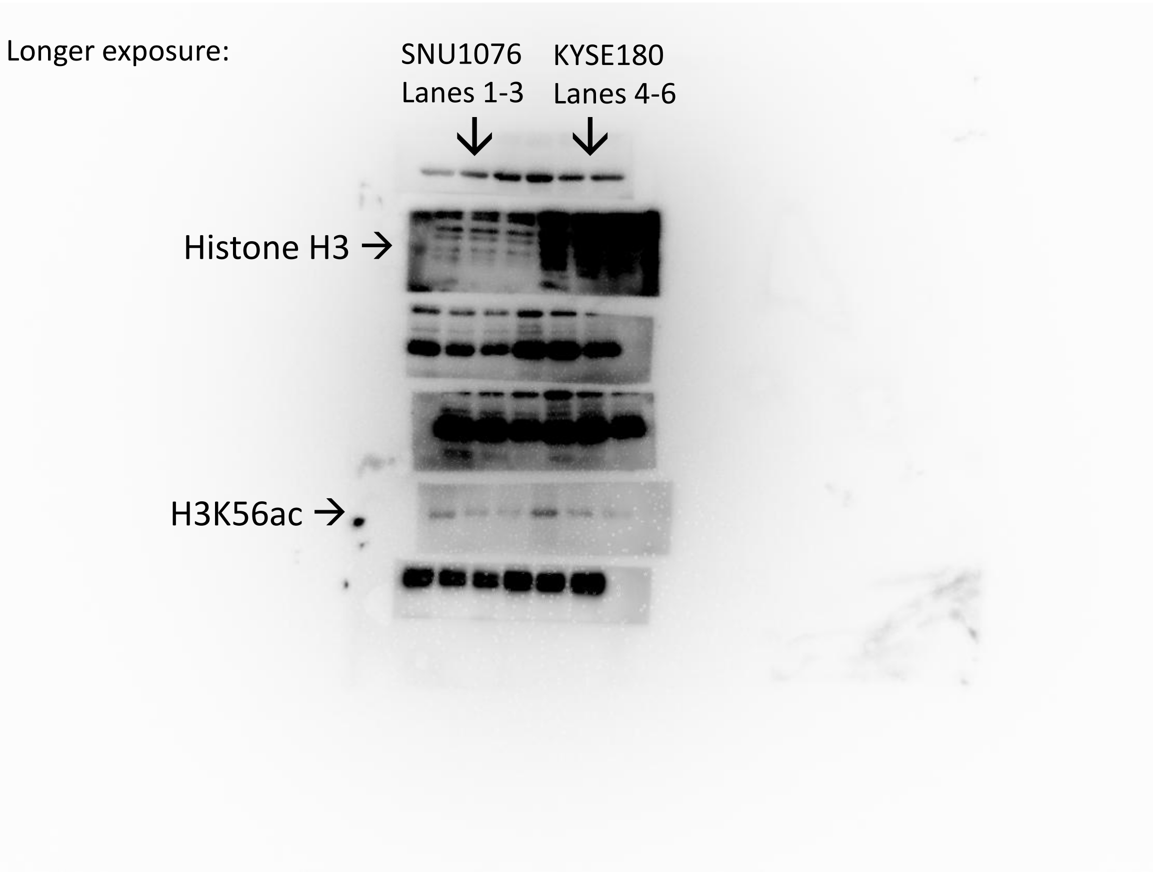
Longer exposure:
SNU1076
Lanes 1-3
KYSE180
Lanes 4-6
Histone H3 →
H3K56ac →

Figure 3A

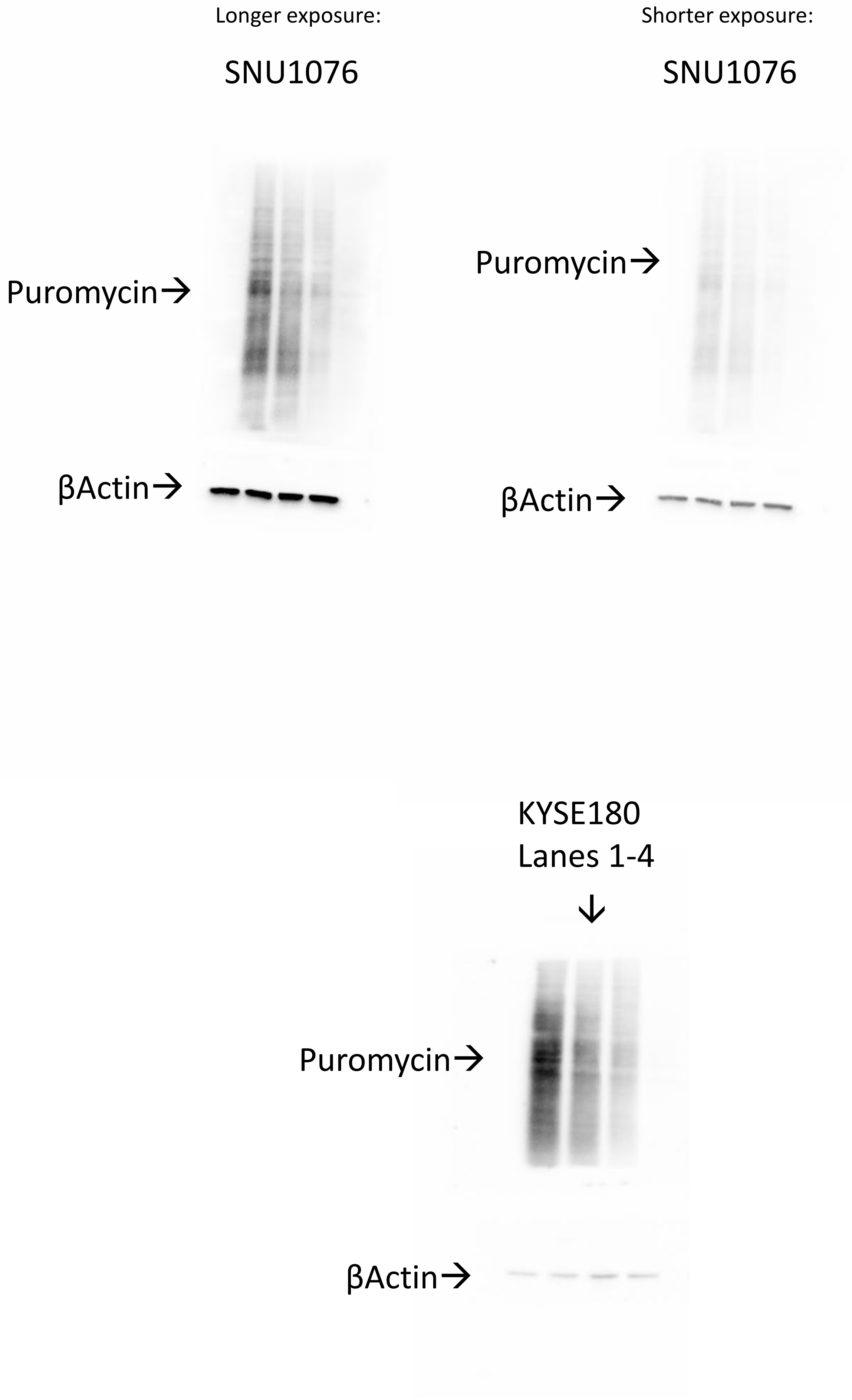
Longer exposure:
SNU1076
Puromycin→
βActin→
Shorter exposure:
SNU1076
Puromycin→
βActin→
KYSE180
Lanes 1-4
Puromycin→
βActin→

Figure 3B

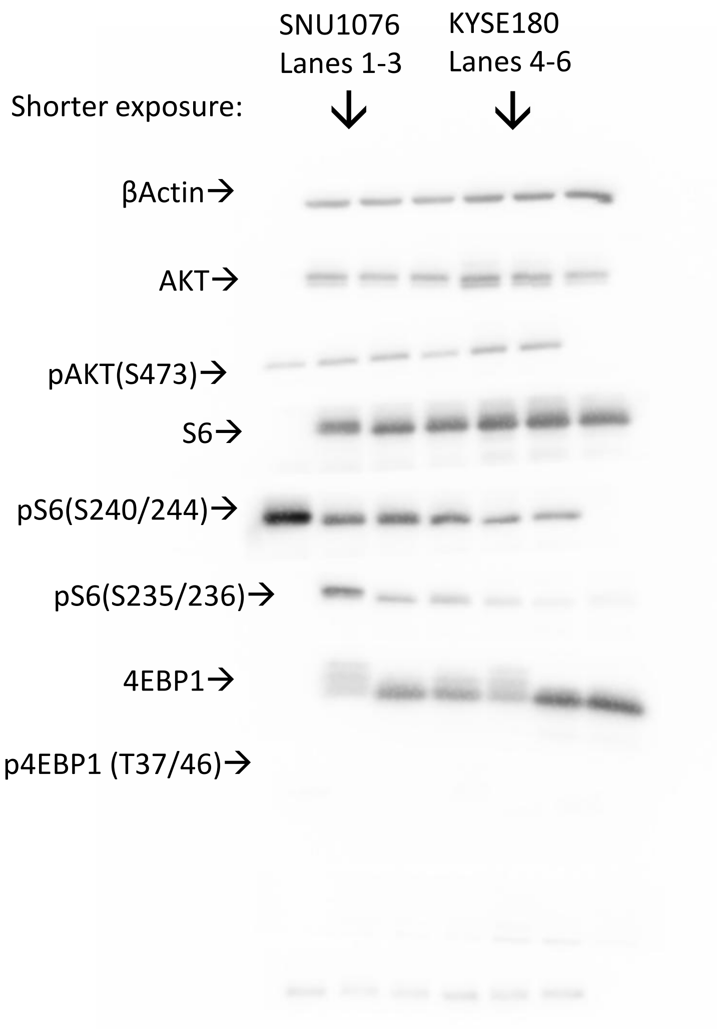
SNU1076
Lanes 1-3
KYSE180
Lanes 4-6
Shorter exposure:
βActin→
AKT→
pAKT(S473)→
S6→
pS6(S240/244)→
pS6(S235/236)→
4EBP1→
p4EBP1 (T37/46)→

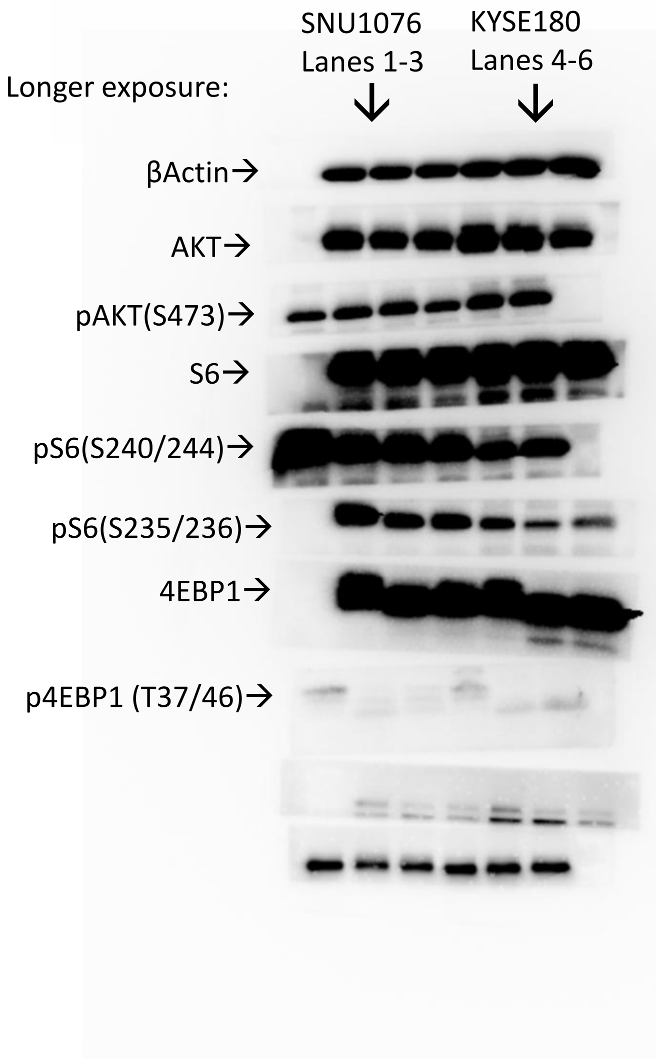
SNU1076
Lanes 1-3
KYSE180
Lanes 4-6
Longer exposure:
βActin→
AKT→
pAKT(S473)→
S6→
pS6(S240/244)→
pS6(S235/236)→
4EBP1→
p4EBP1 (T37/46)→

Figure 3F

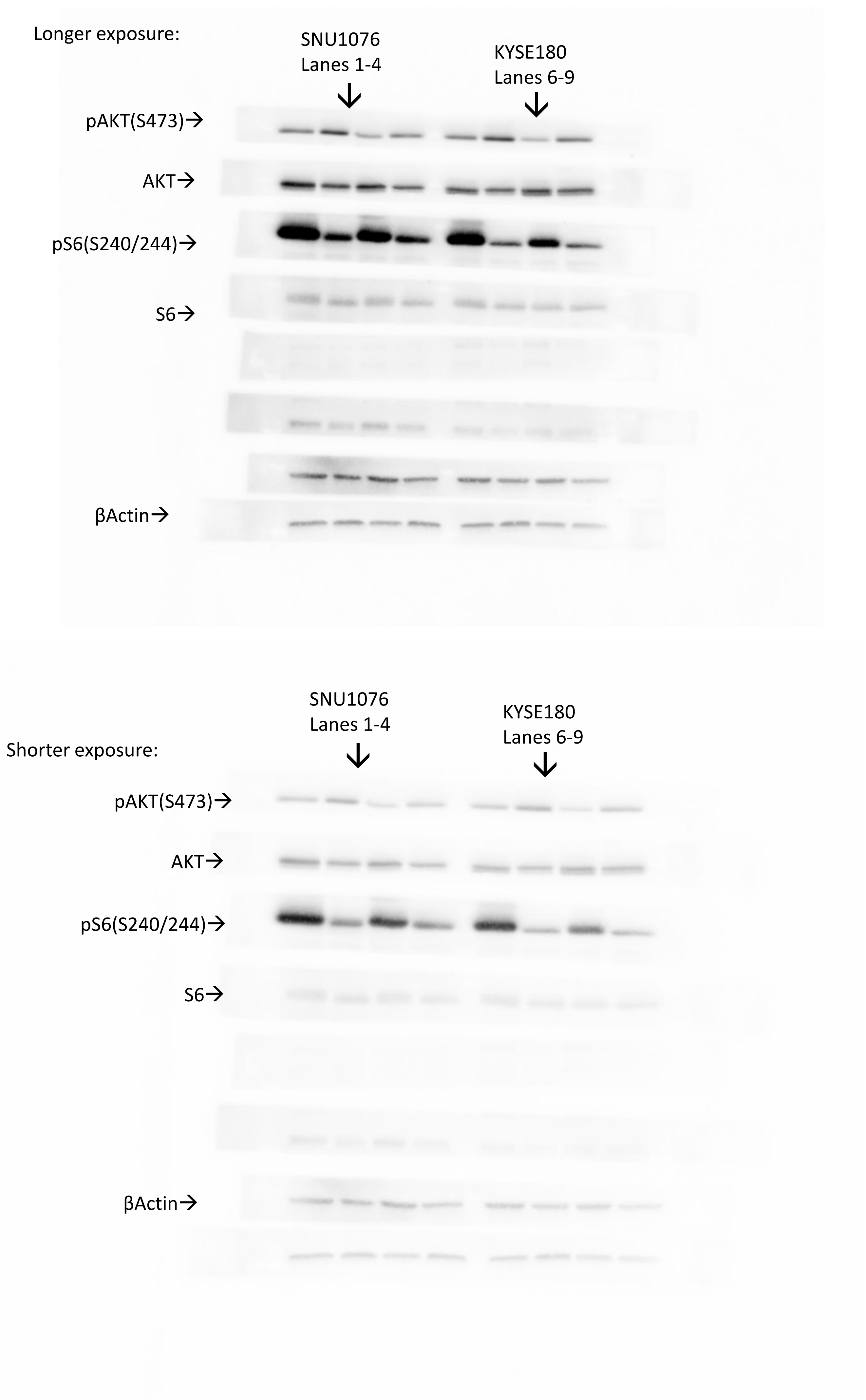
Longer exposure:
SNU1076
Lanes 1-4
KYSE180
Lanes 6-9
pAKT(S473)→
AKT→
pS6(S240/244)→
S6→
βActin→
SNU1076
Lanes 1-4
KYSE180
Lanes 6-9
Shorter exposure:
pAKT(S473)→
AKT→
pS6(S240/244)→
S6→
βActin→

Figure 4A

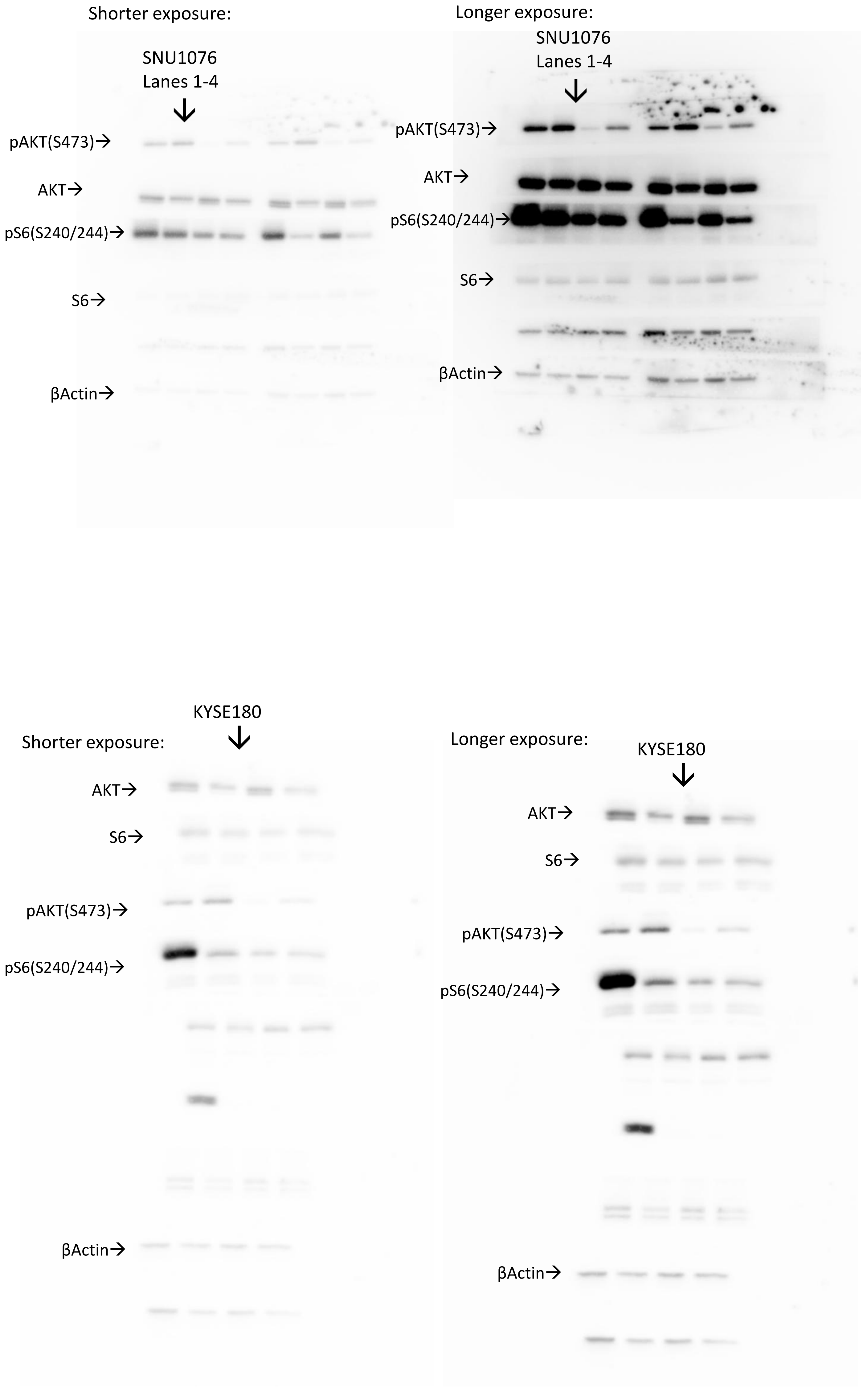
Shorter exposure:
SNU1076
Lanes 1-4
pAKT(S473)→
AKT→
pS6(S240/244)→
S6→
βActin→
Longer exposure:
SNU1076
Lanes 1-4
pAKT(S473)→
AKT→
pS6(S240/244)→
S6→
βActin→
KYSE180
Shorter exposure:
AKT→
S6→
pAKT(S473)→
pS6(S240/244)→
βActin→
Longer exposure:
KYSE180
AKT→
S6→
pAKT(S473)→
pS6(S240/244)→
βActin→

Supplementary Figure 2C

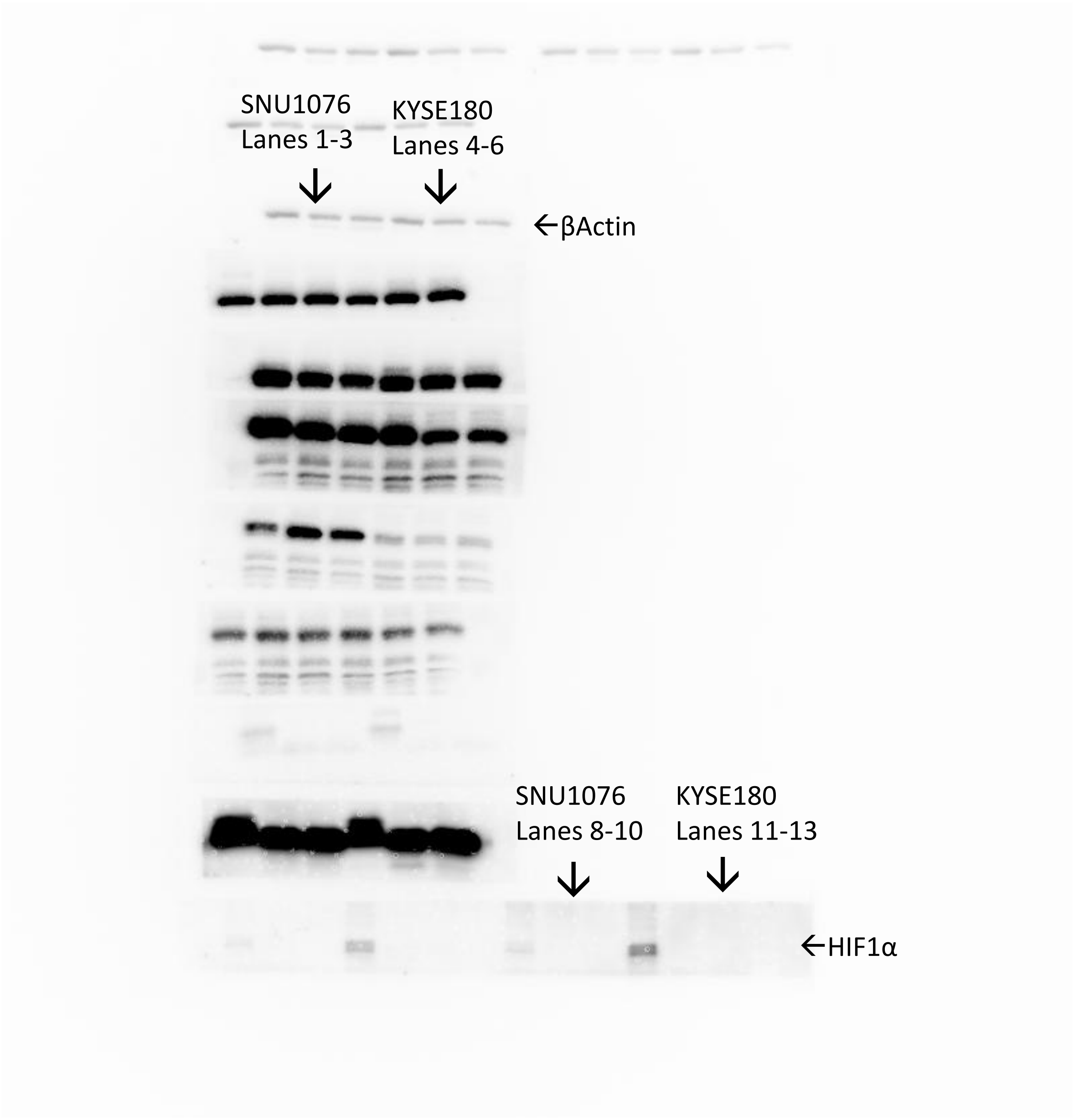

Supplementary Figure 3A

Longer exposure:

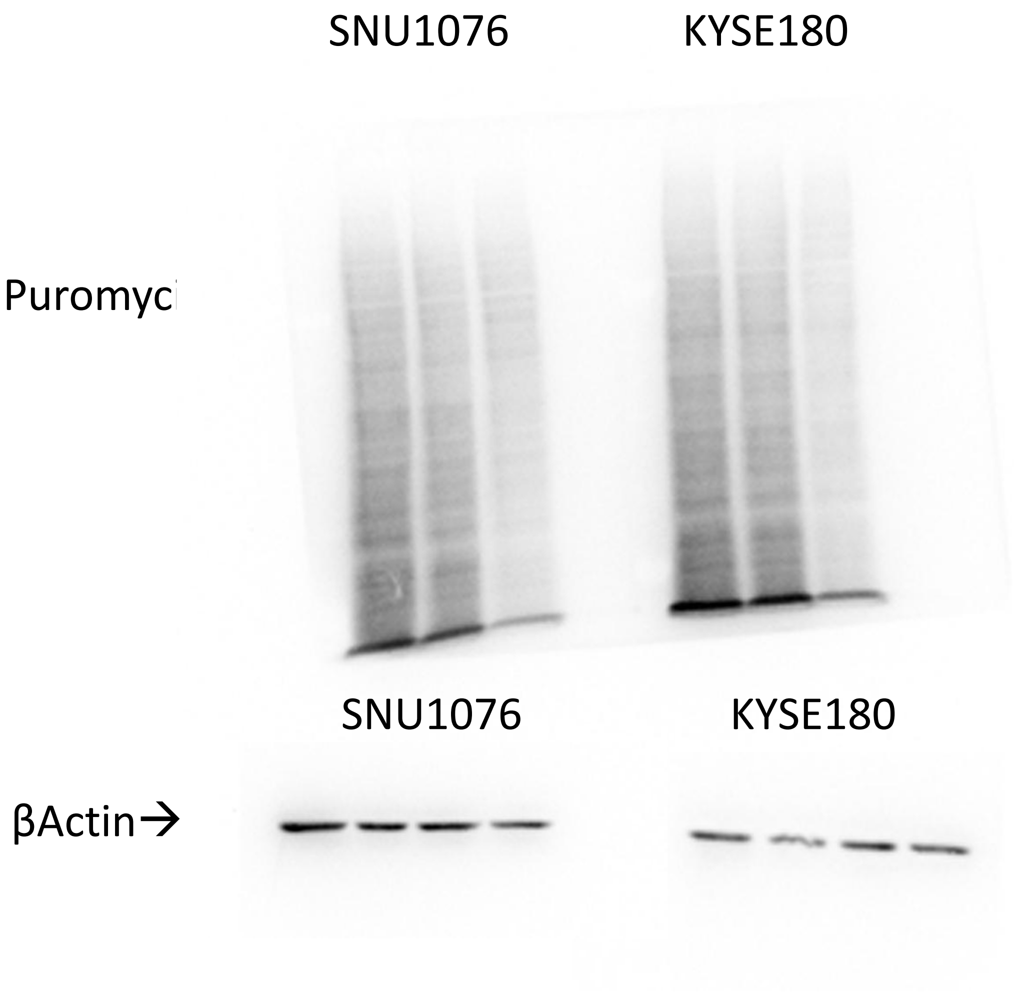

Supplementary Figure 3B – SNU1076

Longer exposure:

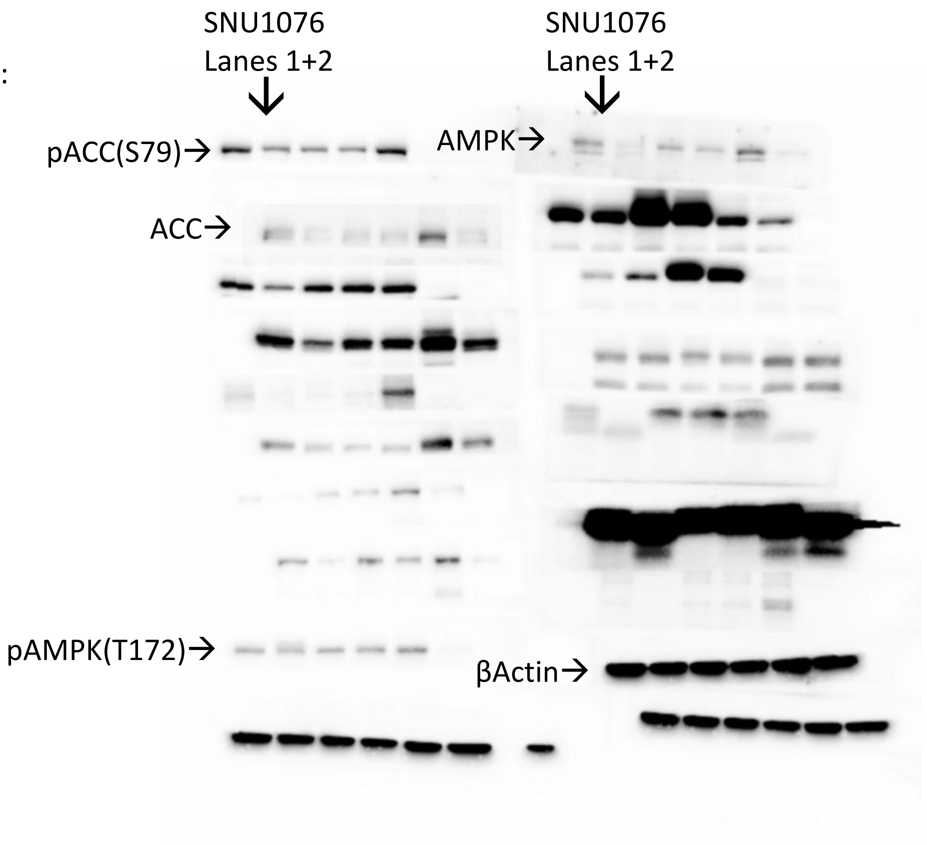

Shorter exposure:

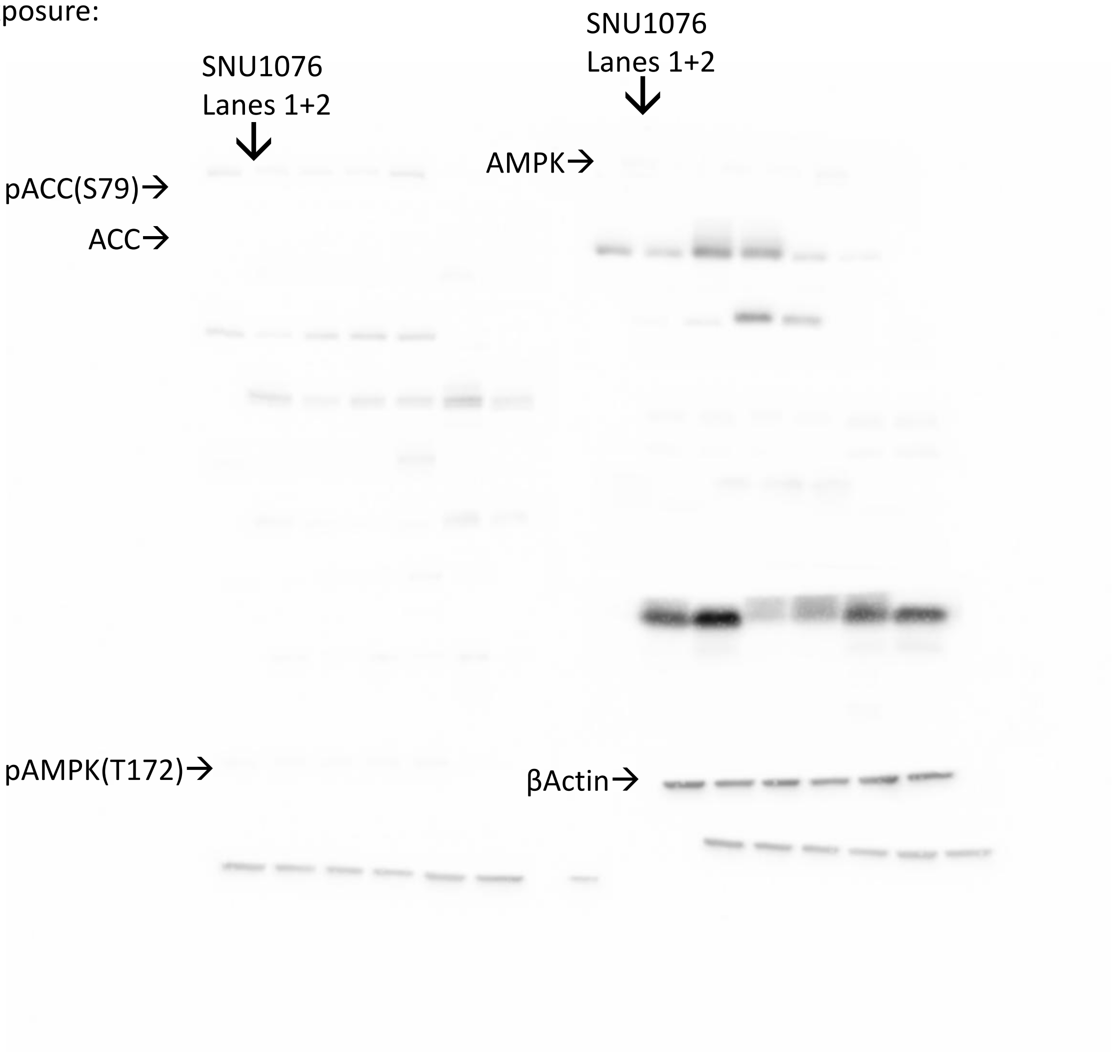

Supplementary Figure 3B-KYSE180

Shorter exposure:

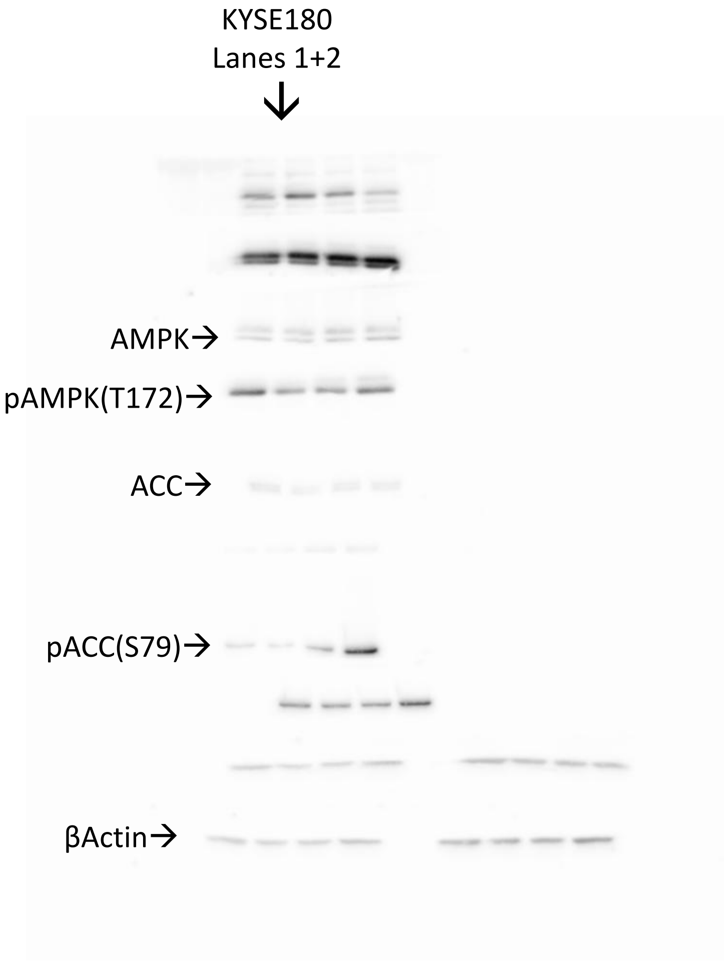

Longer exposure:

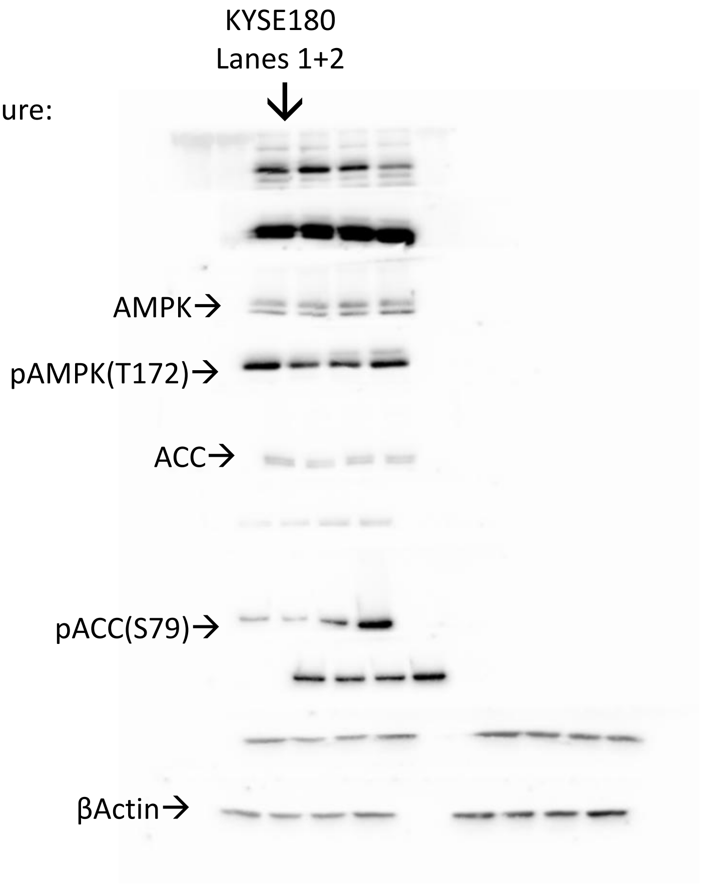

Supplement: Supplementary file 6 — Original Data [file 41419_2025_7959_MOESM6_ESM.pdf]
